# A multi-scale supervised contrastive framework for cross-domain soybean disease classification using leaf and UAV imagery

**DOI:** 10.1038/s41598-026-56162-9

**Published:** 2026-06-02

**Authors:** Shreeya Smita Mohanty, Vaibhavv Maheshwari, Prakash K. Aithal, Roshan David Jathanna

**Affiliations:** Manipal Institute of Technology, Manipal Academy of Higher Education, Manipal, India

**Keywords:** Soybean disease classification, Multi-scale learning, UAV imagery, Leaf-level imaging, Supervised contrastive learning, Cross-domain feature alignment, Domain generalization, Convolutional neural networks (CNNs), Vision transformers (ViTs), Ensemble learning, Grad-CAM, Precision agriculture, Sustainable development goal 2, Zero Hunger, Sustainable development goal 12, Responsible consumption and production, sustainable agriculture crop health monitoring, Computational biology and bioinformatics, Engineering, Mathematics and computing

## Abstract

Accurate and scalable soybean crop health monitoring remains a major challenge in precision agriculture due to environment variability, inconsistent lighting conditions, and significant differences between the ground-level leaf imagery and UAV-based aerial imagery. Most existing deep learning approaches treat these two sensing modalities separately without properly exploring cross-scale feature transferability or measuring the domain gap that exists between the sensing scales. As a result, developing unified and deployment-ready crop health monitoring systems that can effectively leverage the more accessible leaf-level datasets, collected without specialized equipment or regulatory constraints, to improve UAV-scale inference remains difficult. In order to address this limitation, we propose a multi-scale soybean crop health assessment framework that integrates ground-level leaf imagery and UAV-based aerial imagery from the MH-SoyaHealthVision dataset across four health conditions, which include Healthy, Mosaic Virus, Pest attack, and Rust. CLAHE, Gray-World color constancy correction, and illumination normalization is incorporated into a structured pre-processing pipeline and further applied to reduce illumination bias and enhance cross-domain feature consistency. Six deep learning backbones were comprehensively evaluated for leaf-level classification, with MaxViT and ConvNeXt achieving the best performance. Their static weighted ensemble further improved accuracy to 87.08%. Cross-scale evaluation showed that zero-shot leap-to-UAV transfer achieved only 40% accuracy, thus highlighting the presence of a substantial domain shift. Fine-tuning improved UAV classification performance to about 97%, while a supervised contrastive learning framework specifically designed for cross-scale feature alignment further increased accuracy to approximately 98% with better convergence stability. Feature embedding analysis using PCA, t-SNE, and silhouette metrics demonstrated considerable improvements in inter-class separability (0.59 vs. 0.19) and reduced domain discrepancy (0.0336 vs. 0.114) under contrastive learning. These findings suggest that supervised alignment can generate more class-discriminative representations with lower cross-scale domain discrepancy, making them more suitable for scalable multi-scale cross-health monitoring.

## Introduction

Soybean (*Glycine max L.*) is a leguminous crop contributing largely towards global oilseed production, livestock feed supply, and plant-based protein demand, thus showing its heavy economical significance. However, it is also highly vulnerable to biotic environments such as mosaic virus, rust, and pest infestation, as well as abiotic stress factors, which include drought, nutrient imbalance, and genetic structural variations, which can significantly reduce the quality. Traditionally, farmers rely on manual crop scouting methods to verify field conditions. However, this approach is time consuming, subjective and sometimes unable to capture variability across large agricultural fields. As a result the development of scalable and multi resolution crop monitoring systems has become a topic of research in precision agriculture.

Recent developments in unmanned aerial vehicles (UAVs), platforms, multispectral imaging, and deep learning have reshaped agricultural phenotyping and disease monitoring. However, even after such rapid progress, the existing research still remains largely distributed across spatial scales, focusing either on leaf-level disease or UAV-based field-level monitoring, without any attempt to systematically bridge these scales or investigate whether features learned at one level can generalize into another. This limitation motivates the present work.

From a sustainability perspective, this research also contributes to the United Nations Sustainable Development Goals (SDGs), particularly SDG 2: Zero Hunger and SDG 12: Responsible Consumption and Production. Early detection and prediction of soybean leaf diseases enable farmers to take timely preventive measures, thereby reducing crop losses and improving yield stability, which supports global food security. Furthermore, accurate disease prediction enables targeted treatment strategies and reduces excessive pesticide use, promoting environmentally sustainable agricultural practices and responsible resource utilization.

### UAV-based crop health monitoring

UAV-based remote sensing has emerged as a powerful tool for high-throughput crop monitoring due to its flexible deployment, high spatial resolution, and multi-sensor compatibility. While large scale UAVs imagery datasets like MSUAV500K^1^ provide standard benchmarks for model development, foundational soybean UAV datasets and integrated leaf-aerial resources have been introduced to support multimodal research^2^,

Remote sensing along with algorithmic intelligence has been recognized very useful for modern precision agriculture^3^. Researchers have explored various data fusion strategies for UAV-based crop monitoring^4^, including multisensor fusion for nitrogen estimation^5^. In soybean-specific applications, UAV spectral responses have been applied for drought stress analysis and biomass prediction^6^, multimodal drought tolerance evaluation^7^, biochemical trait mapping^8^, maturity monitoring^9^, and multi-source biomass estimation^10^. Alongside disease monitoring using drone-mounted multi-spectral imaging^11^ and hyperspectral severity classification technique^12^, other works have investigated irrigation-stage special modeling for yield forecasting^13^.

Beyond disease detection, UAV platforms have been extensively used for estimating chlorophyll content estimation^14,15^, generating canopy height models through LiDAR and point cloud analysis^16,17^, integrating soil nitrogen information^18^, estimating leaf area index (LAI)^19,20^, and moisture analysis through multi-source feature fusion^21^. These studies show the significant potential of UAV-based phenotyping. However, most approaches operate at aerial level and do not explore whether knowledge learned from fine grained leaf-level imagery can improve UAV-based analysis.

### Deep learning for leaf-level disease classification

In parallel to the advances of UAV, deep learning has revolutionized the recognition of plant diseases on the leaf scale. CNN-based frameworks have shown strong performance in soybean disease classification tasks^22–25^ achieving high accuracy under controlled conditions. Lightweight architectures and ensemble techniques have been proposed to improve computational efficiency and robustness for real-life deployment^26^. Comprehensive surveys in this domain confirm the effectiveness of deep learning in crop disease detection while also highlighting generalization challenges in field environments^27,28^.

More recently, hybrid CNN-Transformer architecture have gained attention due to their ability to capture both fine grained local texture and global contextual dependencies^29–32^. Compared to traditional CNN backbones, these models offer superior representation capacity. Additionally, diffusion-based generative models have been explored for data augmentation and robustness enhancement, helping mitigate issues related to limited or imbalanced datasets^33,34^.

Despite these methods achieve high leaf-level classification accuracy, they typically operate under single-domain assumptions. The critical question of whether fine-grained disease representations learned at leaf level can generalize to UAV-scale imagery remains largely unanswered.

### Cross-domain learning and feature alignment challenges

A major unresolved challenge in agricultural AI is cross-domain and cross-scale generalization. Leaf-level images and UAV imagery differ in viewpoint, spatial resolution, illumination conditions, background clutter, and canopy aggregation.

Several studies have attempted to address related issues through advanced feature fusion frameworks. For example, approaches like integrating transfer learning and attentive convolutional recurrent models have been proposed for crop health monitoring^35^. Rather than explicitly aligning representations across spatial scales, these methods were proposed to improve performance within aerial imagery domains. Insights into representation learning behavior in transformer architectures further emphasize the importance of robust embedding alignment for domain adaptation^36^.

Despite the heavy usage of transfer learning, most agricultural studies still rely on fine-tuning strategies. Quantitative evaluation of inter-class separability and domain invariance still remain rare. Additionally, systematic analysis of zero-shot transfer performance between the leaf-level and UAV-based imagery is largely absent in soybean research.

### Explainable AI in agricultural imaging

Interpretability has become increasingly important as AI-driven systems have become more prevalent in agriculture. Visualization techniques such as Grad-CAM and SHAP have been applied to crop disease detection to get transparency in model decision-making^37^. While these models exhibit high transparency, their applications are usually restricted to the single spatial scale. Developing multiscale explainability, represents an important and unexplored direction in agricultural research, where attention maps are usually validated at both leaf and UAV levels.

### Overview of the proposed framework and contributions

The proposed framework combines systematic backbone benchmarking, ensemble learning, cross-domain transfer evaluation, supervised contrastive learning, and embedding-level feature analysis. First, comprehensive image pre-processing techniques, including CLAHE, square padding, Gray-World color constancy correction, illumination normalization, and standard resizing were evaluated to reduce illumination bias and preserve structural consistency. Second, an extensive backbone comparison was conducted across MobileNet, DenseNet, InceptionNet, EfficientNet, ConvNeXt, and MaxViT. Experimental results revealed a consistent performance progression:

MobileNet < DenseNet < InceptionNet < EfficientNet < ConvNeXt < MaxViT.

Among these ConvNeXt and MaxViT emerged as the top-performing architectures. A weighted ensemble combining of ConvNeXt and MaxViT achieved the highest overall accuracy. Notably, attention-based weighted ensembling did not outperform static weighted averaging, suggesting limited generalization benefits from dynamically learned ensemble weights in this context.

Third, cross-scale robustness was systematically evaluated using a progressive three-stage protocol. Zero-shot transfer was used as a domain gap diagnostic to establish a quantitative lower bound for cross-skill transferability, and then fine-tuning acted as a strong domain adaptation baseline, showing that the leaf-level representations still preserve transferable low-level features while also highlighting the remaining domain discrepancy after adaptation. Finally, supervised contrastive learning was introduced to directly address this residual discrepancy by organizing the embedding space for cross-skill generalization, which further enhanced convergence stability.

Embedding analysis using PCA and t-SNE, combined with silhouette score evaluation, quantitatively validated feature alignment improvements. Compared to fine-tuning (class silhouette *≈* 0.19; domain silhouette *≈* 0.114), the contrastive model achieved measurably higher inter-class separability (*≈* 0.59) and reduced domain discrepancy (*≈* 0.0336), demonstrating strong domain invariance.

The primary contributions of this work are motivated by the three gaps identified in Section 2.6 namely scale fragmentation, limited cross-domain evaluation, and lack of sufficient multi-scale interpretability and are summarized as below:

- *Systematic backbone benchmarking* To address scale fragmentation, six deep learning architectures were evaluated under identical conditions on leaf-level imagery, establishing a reproducible baseline for soybean disease classification.
- *Progressive cross-scale transfer protocol* To reduce the leaf-to-UAV domain gap, a three-stage evaluation comprising zero-shot transfer, fine-tuning, and supervised contrastive learning provides the first quantitative assessment of domain shift magnitude in multi-scale soybean monitoring.
- *Supervised contrastive alignment framework* To address the absence of feature-level alignment validation, a supervised contrastive learning framework explicitly structures the embedding space for cross-scale generalization.
- *Quantitative embedding validation* Inter-class separability and domain discrepancy were evaluated using silhouette metrics, PCA, and t-SNE, showing objective evidence of alignment quality.
- *Ensemble strategy analysis* Static weighted averaging is demonstrated to outperform attention-based dynamic fusion under moderate dataset sizes, offering a practically useful finding for agricultural AI practitioners.
- *Multi-scale interpretability* Grad-CAM visualizations validated at both leaf and UAV scales confirmed that model predictions were focused on biologically meaningful disease regions.

## Literature review

### UAV-based soybean monitoring and spectral phenotyping

Unmanned Aerial Vehicles (UAVs) have drastically transformed precision agriculture by enabling high-resolution, rapid, and non-destructive monitoring of crop field conditions. The combination of algorithms with remote sensing platforms has created a strong foundation for data-driven agriculture decision-making^3^. Multispectral and RGB imagery for agricultural applications have been provided by large-scale UAV datasets, such as MSUAV500K^2^. Additionally, the MH-Soya Healthvision dataset^1^ introduced in our research enables multiscale crop health assessment combining the UAV imagery with ground-level soybean leaf images.

UAV-based multispectral and hyperspectral sensing has been highly used in soybean phenotyping tasks, including maturity detection, chlorophyll estimation, biomass prediction, and drought tolerance evaluation. Hyperspectral UAV imagery was used for mapping soybean maturity and biochemical traits^8^, while deep learning-based UAV monitoring frameworks have shown effectiveness for soybean maturity prediction^9^. Similarly, with the help of fusion of multiple UAV dataset soybean biomass estimation has been improved with the combination of machine learning algorithms^10^. UAV spectral analysis have also helped in validating the yield prediction using canopy architecture and spectral mechanisms^13^.

Using UAV-mounted multispectral systems, disease-specific monitoring have shown promising results in detecting bacterial blight and other canopy-level stress indicators^11^ and hyperspectral response analysis has further led to severity-level classification for soybean diseases using machine learning techniques^12^. UAV-derived texture indices were used to estimate soybean chlorophyll content^22^, while multi-scale chlorophyll monitoring frameworks have been developed for vertical canopy analysis^23^.

Other than spectral analysis, UAV-based LiDAR has been used for canopy height estimation using regression and classification strategies^24^ while lightweight UAV systems combined with deep learning have also been utilized for yield estimation and lodging discrimination^25^. Furthermore, integration of UAV multispectral imaging with ground-truth soil nitrogen content enhanced precision in yield estimation models^26^. Importance of feature-level fusion in soybean phenotyping was shown by estimation of leaf area index (LAI) and biomass using UAV multispectral imagery^27^ and multisource UAV feature fusion has also been applied for soybean leaf moisture estimation^38^.

Recent studies have introduced graph-inspired semantic compression frameworks combined with enhanced YOLO architectures for resource-efficient UAV-based leaf disease detection, achieving strong object localization performance in paddy rice agronomy^39^. While such approaches advance UAV-based detection efficiency, they are limited to single crop domain and do not explore cross-scale feature transferability between ground-level and aerial sensing modalities, which remains an important research gap addressed in the present work.

Overall, these studies demonstrate the effectiveness of UAV-based spectral and structural analysis for canopy-level soybean monitoring. However, a major limitation is that most UAV-based approaches primarily focus on regression tasks such as yield and biomass estimation, while giving comparatively less attention to categorical disease classification and the possibility of enhancing aerial representations through knowledge transfer from finer-scale leaf imagery.

### Deep learning for soybean leaf disease detection

Deep learning has been seen as a very useful approach for plant disease classification. CNN-based architectures such as DenseNet, Inception, and MobileNetV2 have demonstrated strong performance in soybean leaf disease recognition tasks^29^ whereas region-based detection frameworks combining YOLO for leaf extraction followed by ResNet-50 classification have further improved localization and classification accuracy^30^.

Automated CNN-based soybean leaf disease detection pipelines have also been proposed to improve real-time applicability^31^. Generative Adversarial Networks (GANs) were used in data augmentation to address dataset imbalance and improve generalization performance^32^. Lightweight ensemble strategies incorporating Choquet fuzzy integration have further enhanced robustness while maintaining computational efficiency^33^. Deep convolutional networks integrated with the Internet of Things have expanded the deployment possibilities in practical agricultural environments^21^.

Even though these approaches achieve high accuracy on leaf-level datasets, they usually operate under controlled imaging conditions and single-scale assumptions but cross-scale transfer from leaf-level training to UAV canopy imagery remains largely unexplored.

### Hybrid CNN-transformer architectures and lightweight models

Recent progress in plant disease recognition heavely focused on hybrid deep learning architectures that combines convolutional neural networks and Vision Transformers (ViTs). Extensive survey in studies highlights the growth of deep learning in agricultural disease detection^34,36^. CNN-Transformer hybrid models such as ConvTransNet-S have demonstrated improved feature representation under complex field environments^35^ and similarly, integrated CNN-ViT models have shown enhanced robustness to challenges like background clutter, variations, occlusion and illumination^40^.

The combination of extraction local spatial feature from CNNs with global contextual modeling from transformers significantly improves disease classification stability^14^. Lightweight transformer-based architectures such as MobileH-Transformer also enable real-time leaf disease detection, making them suitable for smart agriculture applications^18^. In additon, attention-based convolutional recurrent neural networks have also been used for crop health monitoring in remote sensing areas, capturing both spatial and temporal relations^17^.

Despite the improvement in representation capacity, the main challenge still remains is that transformation-based architectures have not been systematically evaluated within multi-scale frameworks that integrate both UAV and leaf-level imagery. As a result, their ability to generalize across sensing scales for soil condition monitoring remains largely unexplored.

### Multimodal fusion and cross-domain learning

Multimodal data fusion of UAV has become increasingly important for improving robustness in agricultural vision systems. UAV multispectral fusion combined with physiological and environmental parameters has improved soybean maturity classification performance^41^ and feature-level integration of spectral and structural attributes enhances stability under heterogeneous field conditions^26,27^.

In broader computer vision research, cross-domain representation learning has been studied to improve generalization across diverse datasets. The role of the CLS token in cross-domain few-shot learning shows the importance of embedding alignment in transformer-based architectures^42^. Diffusion-based augmentation combined with transfer learning has been applied to plant disease datasets to improve performance when training data is limited^16,43^.

Despite recent progress in multimodal fusion, the main challenge still remains in effectively aligning feature representations across leaf and UAV sensing scales using supervised contrastive learning. Additionally, soybean research rarely includes quantitative evaluation of domain alignment using embedding-level metrics.

### Explainability and trust in agricultural AI

Explainable AI (XAI) techniques have gained high importance in agricultural decision-support systems. Visualization methods like as SHAP and Grad-CAM are now used to interpret disease classification models and verify biologically meaningful feature learning^15^. These interpretability approaches increases user’s trust and ensures that models focus on disease-relevant regions rather than background artifacts.

### Comparative analysis of existing methods

To clearly state the contributions of the proposed framework within the existing literature, Table 1 presents a systematic comparison of representative studies in soybean disease detection and UAV-based crop monitoring across key aspects such as dataset, imaging modality, model architecture, transfer strategy, and application scenario.

**Table 1:** Systematic comparison of existing methods and the proposed framework.

Study	Crop	Data scale	Modality	Architecture	Transfer strategy	Application
Helmawati et al.^22^	Soybean	Leaf only	RGB	DenseNet, MobileNet	ImageNet fine-tuning	Leaf disease classification
Korade et al.^23^	Soybean	Leaf only	RGB	YOLO + ResNet-50	ImageNet fine-tuning	Leaf detection and classification
Meng et al.^13^	Soybean	UAV only	Multi-spectral	CNN	None	Bacterial blight detection
Jia et al.^35^	Multiple	Leaf only	RGB	CNN-Transformer hybrid	ImageNet fine-tuning	Leaf disease classification
Thai and Le^18^	Multiple	Leaf only	RGB	MobileH-Transformer	ImageNet fine-tuning	Real-time leaf disease detection
Samha et al.^17^	Multiple	UAV only	RGB	CNN-RNN	Transfer learning	Crop health monitoring
Tetila et al.	Soybean	UAV only	RGB	Inception	ImageNet fine-tuning	UAV soybean disease classification
Proposed	Soybean	Leaf + UAV	RGB	ConvNeXt + MaxViT Ensemble	Supervised contrastive cross-scale	Multi-scale disease classification

As shown in Table 1, existing approaches mainly operate at a single spatial scale, focusing independently on either leaf-level disease classification or UAV-based canopy monitoring. To the bets of our knowledge, no prior study has systematically explored cross-scale feature transferability between leaf and UAV imagery for soybean disease classification or quantitatively analyzed the domain gap using zero-shot evaluation and contrastive alignment. These limitations motivate the three primary research gaps discussed in the following subsection.

### Motivation and research gap

Review of the literature shows three primary gaps:

- *Scale Fragmentation* Existing works tend to focus either on leaf-level classification or UAV-based phenotyping, without systematically connecting the two.
- *Limited Cross-Domain Evaluation* Zero-shot transfer performance and quantitative domain alignment metrics are rarely reported.
- *Insufficient Multi-Scale Interpretability* Very few studies validate model explainability across different image resolutions.

To address these challenges, we proposed a multi-scale soybean crop health assessment framework leveraging both ground-level leaf imagery and UAV-based aerial imagery from the MH-SoyaHealthVision dataset. The dataset includes four major soybean health conditions: Healthy, Mosaic Virus, Pest Attack, and Rust.

## Methodology

This section provides the design choices used to make the proposed multi-scale soybean health assessment framework. Although the core components namely ensemble learning, transfer learning, and supervised contrastive learning are already well established, but the specific innovations of this work lie in: (1) the systematic evaluation and selection of complementary backbone architecture for agricultural multi-scale imagery. (2) the development of progressive three-stage cross-scale transfer protocol that measures the extent of domain shift before introducing alignment strategies. (3) an adaptive supervised contrastive learning approach in which leaf and UAV images are jointly sampled within the same mini-batch to explicitly reduce intra-class domain discrepancy across instance scales. And (4), the use of silhouette-based embedding analysis as a quantitative validation tool for cross-scale feature alignment in soybean disease classification.

**Fig. 1:**
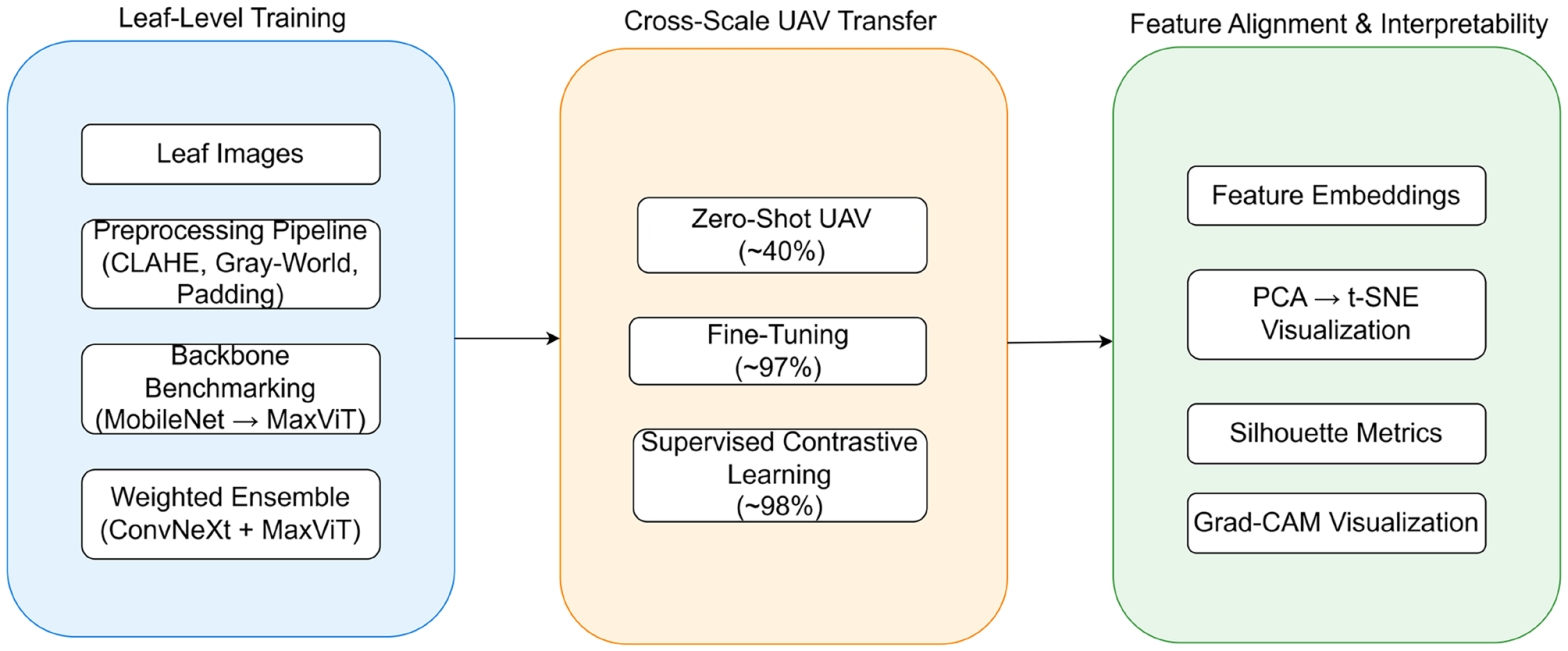
Overview of the proposed multi-scale soybean disease classification framework.

The general architecture of this framework is illustrated in Fig. 1.

### Dataset description

We conducted the experiments using the MH-SoyaHealthVision dataset^2^, which contains both ground-level leaf imagery and UAV-based aerial imagery, both captured under real agricultural field conditions across two seasons (July–November 2023 and July–November 2024) from six village locations spanning two districts of Maharashtra, India: Sangli (Chikhali, Upavale, Hingangaon, and Kadepur) and Satara (Banawadi and Goware). Data acquisition covered multiple plant growth stages including Early Vegetative, Late Vegetative, Reproductive, and Maturity stages, ensuring that disease progression across the crop lifecycle is represented. Disease annotations were carried out with guidance from agricultural experts and university specialists, ensuring reliable labeling across both imaging modalities. Table 2 summarizes the key acquisition characteristics of the dataset.

**Table 2:** MH-SoyaHealthVision dataset acquisition characteristics^2^.

Property	Details
Geographic location	Sangli and Satara districts, Maharashtra, India
Acquisition period	July–November 2023 and July–November 2024
Collection sites	Chikhali, Upavale, Hingangaon, Kadepur (Sangli); Banawadi, Goware (Satara)
Growth stages covered	Early Vegetative, Late Vegetative, Reproductive, Maturity
Leaf image devices	iPhone 14 Plus, Samsung Galaxy F34 5G, OPPO A31
Leaf image resolution	Up to 3072*×*4096 pixels (RGB)
UAV device	DJI Mini 4 Pro (DJI-FC8482)
UAV image resolution	3840*×*2160 pixels (s-RGB)
UAV flight altitude	12–25 meters above field
Annotation method	Expert-guided polygon annotations (leaf); superpixel-based segmentation (UAV)

The UAV dataset annotations were performed using a superpixel-based segmentation workflow as documented in the original dataset publication^2^. UAV images were first preprocessed using HSB-based color segmentation to identify disease-indicative regions, generating binary disease masks where significant color deviations from healthy tissue indicate infection. Simple Linear Iterative Clustering (SLIC) superpixel segmentation was then applied to group spatially coherent pixels into compact regions preserving structural detail. Each superpixel was classified as diseased based on its disease pixel ratio which is the proportion of disease-colored pixels within the superpixel segment relative to total pixels, with superpixels exceeding a predefined threshold classified as diseased and highlighted accordingly. A canopy patch image receives a class label such as “Rust” when rust-indicative superpixels constitute the dominant disease signal across the image, as determined by subject matter experts from agriculture universities who validated all annotations^2^. We acknowledge that the dataset documentation does not specify a precise quantitative threshold for the disease pixel ratio criterion, nor does it define the exact proportion of a canopy patch that must exhibit disease symptoms to qualify for a given class label. This absence of explicit quantitative class criteria represents a dataset-level limitation with implications for cross-scale label correspondence, as discussed in Section Limitations.

The leaf dataset consists of 2835 RGB images captured at close range under natural illumination conditions using smartphone cameras, and contains six disease categories^2^:

- Healthy
- Mosaic
- Rust
- Septoria Brown Spot
- Frogeye Leaf Spot
- Caterpillar and Semi-Looper Pest Attack

Each image is represented as:

$$I \in \mathbb {R}^{H \times W \times 3}$$

where *H* and *W* denote spatial resolution. As cross-scale consistency with the UAV dataset was required, four classes were selected for cross-domain experiments. Septoria Brown Spot and Frogeye Leaf Spot were excluded due to the absence of corresponding UAV annotations.

Table 3 provides the class distribution of the leaf dataset.

**Table 3:** Class distribution of leaf-level dataset.

Class name	Number of images
Healthy	204
Mosaic	707
Rust	852
Septoria brown spot	268
Frogeye leaf spot	222
Caterpillar and semi-looper pest attack	582
Total	2835

**Fig. 2:**
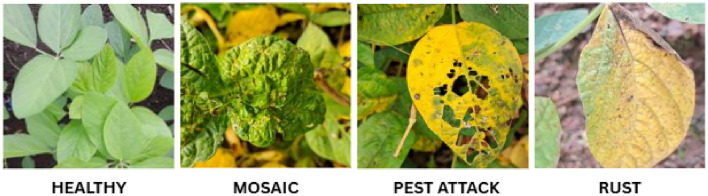
Representative leaf-level samples from the MH-SoyaHealthVision dataset.

Figure 2 presents representative examples illustrating variations in leaf texture, lesion morphology, color distortions, and illumination inconsistencies.

The leaf-level disease classification has been widely explored in literature using deep convolutional networks^22,24,25,41^, but most of that work focuses on single-scale learning without evaluating cross-domain transferability.

The UAV-based dataset contains 2845 aerial images captured using a DJI Mini 4 Pro drone (DJI-FC8482) at flight altitudes maintained between 12 and 25 meters above the field, producing RGB images at 3840*×*2160 pixel resolution. The dataset includes the following classes^2^:

- Healthy
- Mosaic
- Rust
- Caterpillar and Semi-Looper Pest Attack

Regarding label semantics across scales, a leaf-level label such as “Rust” certifies that the individual leaf exhibits rust pustules under close-range imaging, carrying unambiguous individual-level semantics. A UAV canopy label carrying the same term “Rust” denotes dominant disease presence within the canopy patch as determined by expert annotators, representing aggregate-level semantics across multiple plants and leaves in varying health states. This semantic asymmetry is an inherent characteristic of multi-scale agricultural imaging and is consistent with established practice in UAV-based crop disease classification literature^11,13^, where canopy-level labels represent the predominant health condition observable within the aerial field of view. We acknowledge that this asymmetry means cross-scale label correspondence is implicit rather than formally verified, as discussed further in Section Limitations.

Table 4 provides the class distribution of the UAV dataset.

**Table 4:** Class distribution of UAV-based dataset.

Disease/Pest attack	Number of images
Healthy	281
Mosaic	773
Rust	1001
Caterpillar and semi-looper pest attack	790
Total	2845

**Fig. 3:**
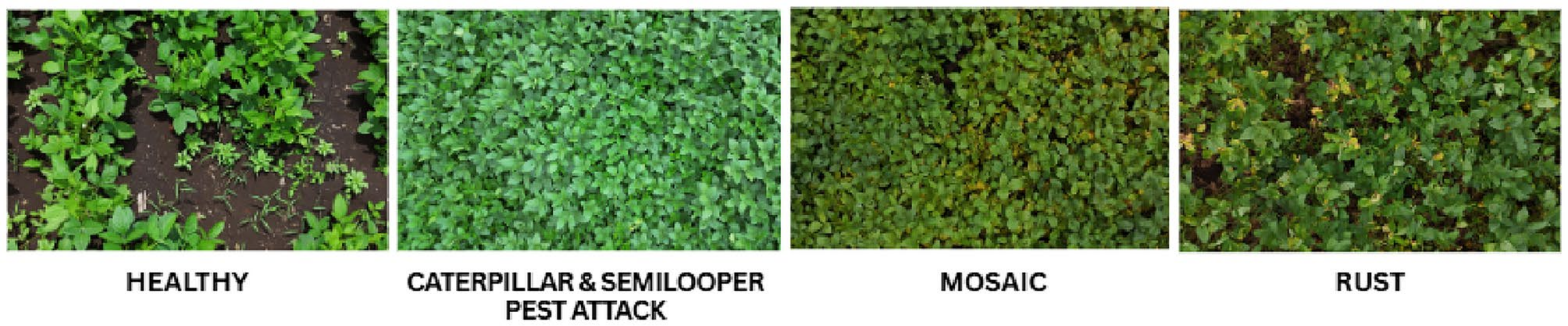
Representative UAV-based aerial samples from the MH-SoyaHealthVision dataset.

Figure 3 shows representative UAV samples highlighting differences in spatial scale and structural patterns compared to leaf images.

#### Scale correspondence and physical sources of domain shift

The difference in scale between leaf-level and UAV-based imagery represents a fundamental source of cross-scale domain shift in this study. Leaf images were captured at close range using smartphone cameras at resolutions up to 3072*×*4096 pixels, where individual disease symptoms such as rust pustules, lesion edges, and chlorotic patches occupy a substantial portion of the image frame, providing fine-grained textural detail at the pixel level. In contrast, UAV imagery was captured at altitudes between 12 and 25 meters, producing 3840*×*2160 pixel images in which individual leaves occupy only a small fraction of the frame, disease lesions may span only a few pixels or become entirely unresolvable, and the field of view encompasses entire canopy regions rather than individual leaf surfaces.

This scale difference gives rise to four distinct physical sources of cross-scale domain shift:

- *Spatial resolution reduction*: Disease symptoms spanning hundreds of pixels in leaf imagery are compressed to a few pixels or sub-pixel regions in UAV imagery, fundamentally altering the textural features available for classification.
- *Viewpoint and perspective change* Leaf images are captured at near-perpendicular close range to individual leaf surfaces, while UAV imagery captures canopy structure from a nadir aerial perspective, introducing significant differences in shape, orientation, and occlusion patterns.
- *Background complexity* Leaf images are captured with relatively controlled framing where the leaf occupies the majority of the frame, whereas UAV imagery contains substantial background clutter including soil, shadows, overlapping canopy layers, and inter-plant variation.
- *Illumination geometry* Smartphone captures under natural diffuse lighting produce relatively uniform illumination across the leaf surface, whereas UAV imagery is subject to directional sunlight, canopy shadow patterns, and atmospheric scattering that introduce spatially varying illumination across the image.

These four physical factors collectively explain the substantial domain shift quantified in our cross-scale transfer evaluation and motivate the need for explicit cross-scale feature alignment strategies.

### Image preprocessing pipeline

When agricultural images are captured under real-world field conditions, they often contain large variation in lighting, color balance, and background noise^27,28^. To improve feature consistency across images, a structured pre-processing pipeline was implemented.

The choice and sequence of each preprocessing stage was carefully designed based on the common degradation factor present in agricultural field imagery, rather than following arbitrary convention. Overall, the pipeline targets three major sources of feature inconsistency, which are illumination variation, environmental color cast, and geometric distortion from aspect ratio variation. CLAHE is applied first to enhance local luminance contrast using the original image before any color correction is introduced. Applying CLAHE after Gray-World correction could unintentionally amplify artificially shifted color channels instead of the true scene seen luminance. Gray-World correction is then applied because its neutral scene assumption that the average scene reflectance is achromatic becomes more reliable after local illumination variation have already been normalized. Then square padding is performed before resizing to avoid any kind of distortion of disease lesion shapes that could occur during direct non-square resizing. Finally, ImageNet normalization is applied after all preprocessing operations are done so that normalization is performed on fully corrected pixel values consistent with the pre-trained backbone input distribution. Applying normalization earlier would have altered the pixel range expected by later preprocessing steps, reducing the effectiveness of both CLAHE and gray world correction.

The pipeline consists of the following stages:

**Stage 1:** LAB-based Contrast Limited Adaptive Histogram Equalization (CLAHE)

CLAHE is applied in the CIELAB color space to improve the local contrast while preserving chromatic information. Operation like enhancement is performed only on the L channel so that the luminous correction remains separated from the color information, which is especially important for soybean disease classification where color acts as the key diagnostic feature. For example, yellow discoloration from mosaic virus, brown rust, pustules, and healthy green tissue must remain visually distinguishable after pre-processing. Unlike the standard histogram equalization, CLAHE performs localized histogram equalization within smaller tile range (Clip Limit = 2.0; Tile Grid = 8*×*8). This allows low-contrast lesion regions to be enhanced without excessively amplifying an already well-illuminated areas, thus making it more suitable for heterogeneous agricultural field imagery^27^.

The RGB image is converted to LAB:

$$I_{LAB} = \text {RGB2LAB}(I)$$

CLAHE is applied to the luminance channel *L*:

$$L' = \text {CLAHE}(L)$$

The image is reconstructed:

$$I' = \text {LAB2RGB}(L', a, b)$$

**Stage 2:** Gray-World Color Constancy

To reduce the color imbalance caused by environmental lighting we make use of Gray-World correction. This method assumes that the average reflectance of the scene is achromatic, and scales each color channel accordingly to correct for illumination-induced color cast. This specifically addresses the chromatic variability introduced across the six collection sites of the MH-SoyaHealthVision dataset, which were acquired across two seasons under varying atmospheric and lighting conditions^2^:

$$R' = R \cdot \frac{\mu }{\mu _R}, \quad G' = G \cdot \frac{\mu }{\mu _G}, \quad B' = B \cdot \frac{\mu }{\mu _B}$$

where

$$\mu = \frac{\mu _R + \mu _G + \mu _B}{3}$$

This normalization reduces illumination bias and stabilizes chromatic features across images captured under different environmental conditions.

**Stage 3:** Square Padding and Normalization

Square padding is applied before resizing to preserve the original aspect ratio of both leaf and UAV images. Direct resizing of non-square images to 224*×*224 pixels introduces anisotropic geometric distortion that alters the shape and spatial proportions of disease lesions, for example, circular rust pustules would become elliptical and elongated lesion patterns would be compressed thereby potentially affecting texture and shape features used by convolutional layers for classification. Square padding with zero-value borders preserves original geometric relationships while ensuring fixed-size network input compatibility. ImageNet normalization is then applied last:

$$I_{norm} = \frac{I - \mu _{ImageNet}}{\sigma _{ImageNet}}$$

**Fig. 4:**
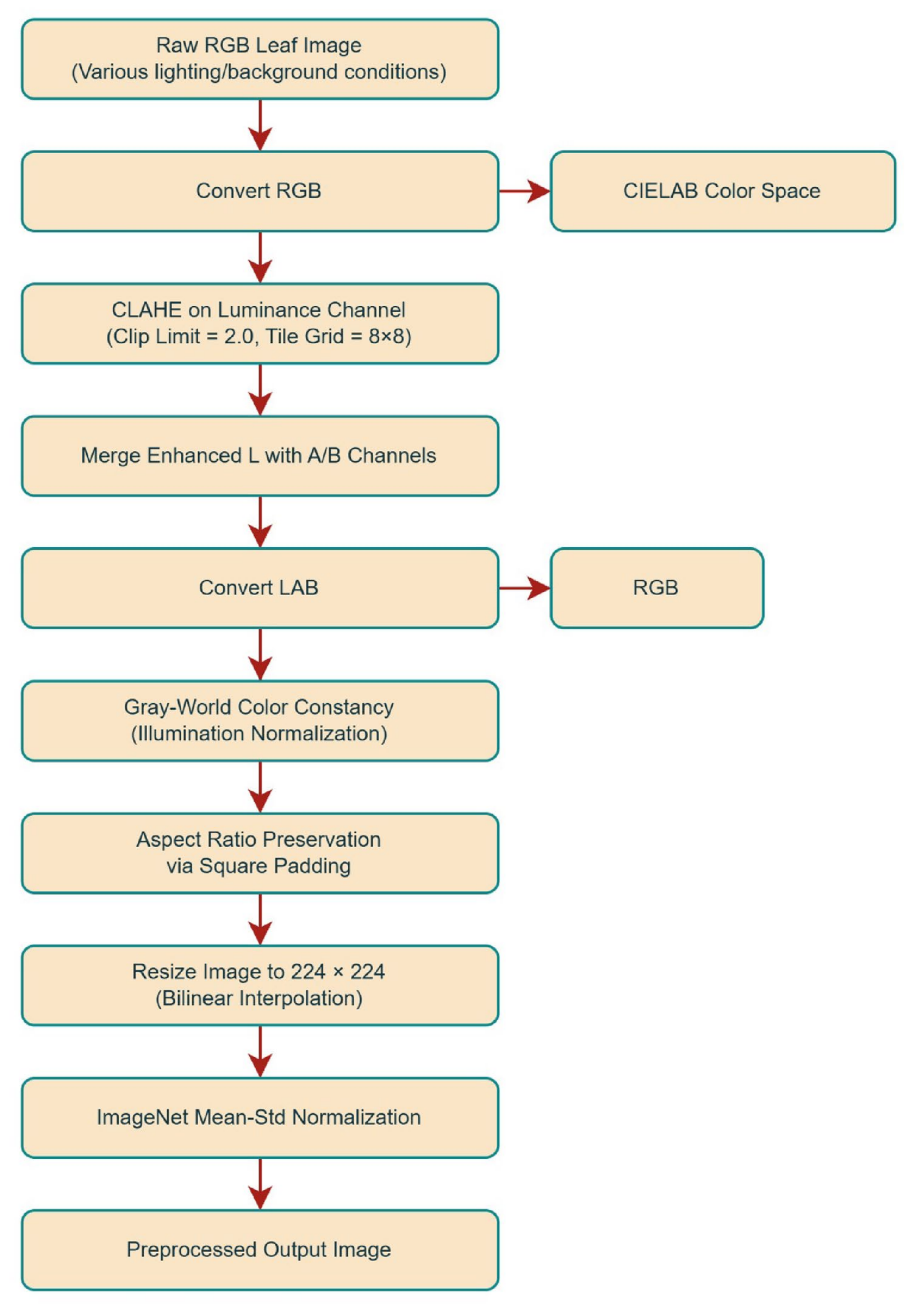
Proposed image preprocessing pipeline for soybean leaf and UAV imagery comprising four sequential stages: (1) RGB to CIELAB conversion followed by CLAHE on the luminance channel (Clip Limit = 2.0, Tile Grid = 8*×*8) to enhance local contrast while preserving chromatic integrity, (2) LAB to RGB reconstruction and Gray-World color constancy correction for illumination normalization, (3) aspect-ratio-preserving square padding, and (4) bilinear resizing to 224*×*224 pixels followed by ImageNet mean-std normalization.

**Fig. 5:**
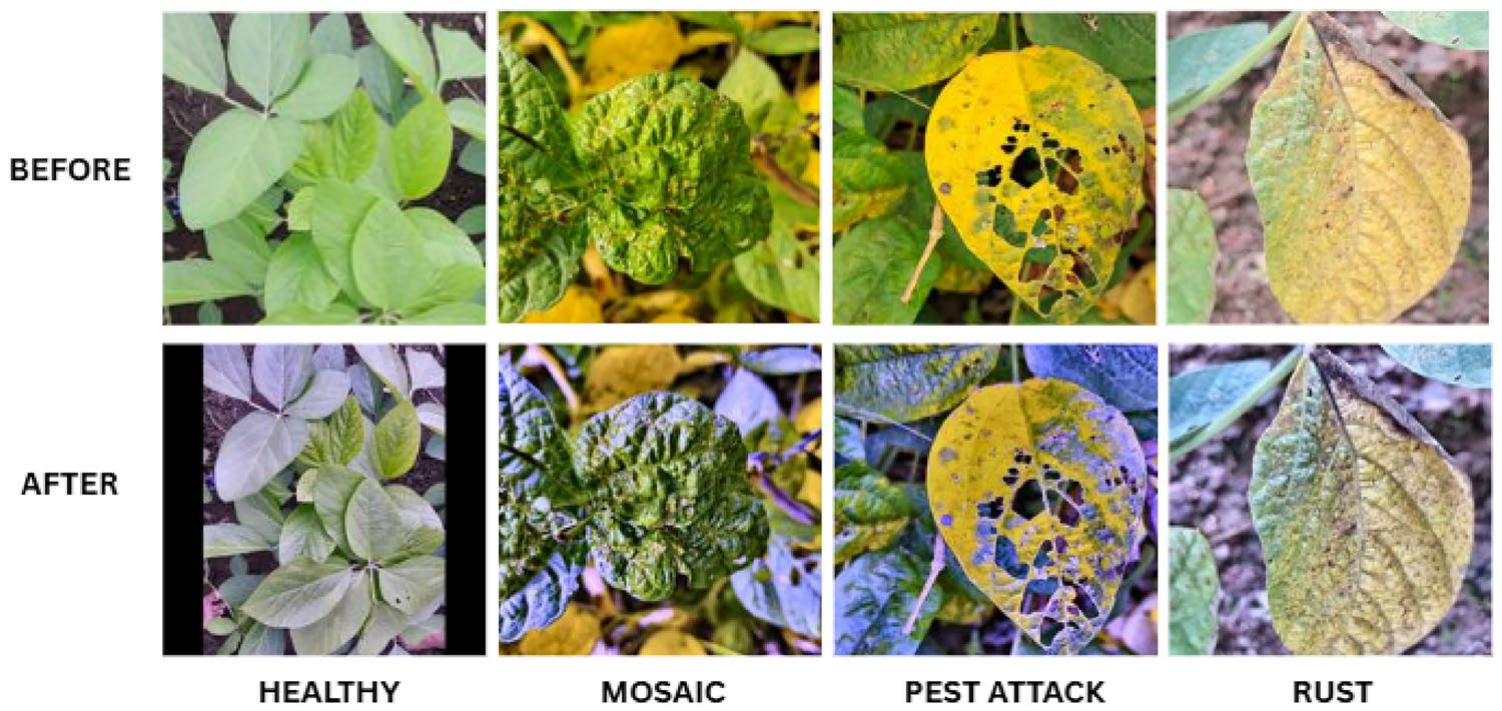
Representative raw and preprocessed soybean leaf samples illustrating the effect of the proposed preprocessing pipeline (LAB-based CLAHE, Gray-World correction, square padding, and normalization).

Figure 4 illustrates the complete preprocessing pipeline and Fig. 5 shows representative raw and preprocessed image pairs.

### Leaf-level backbone benchmarking

**Fig. 6:**
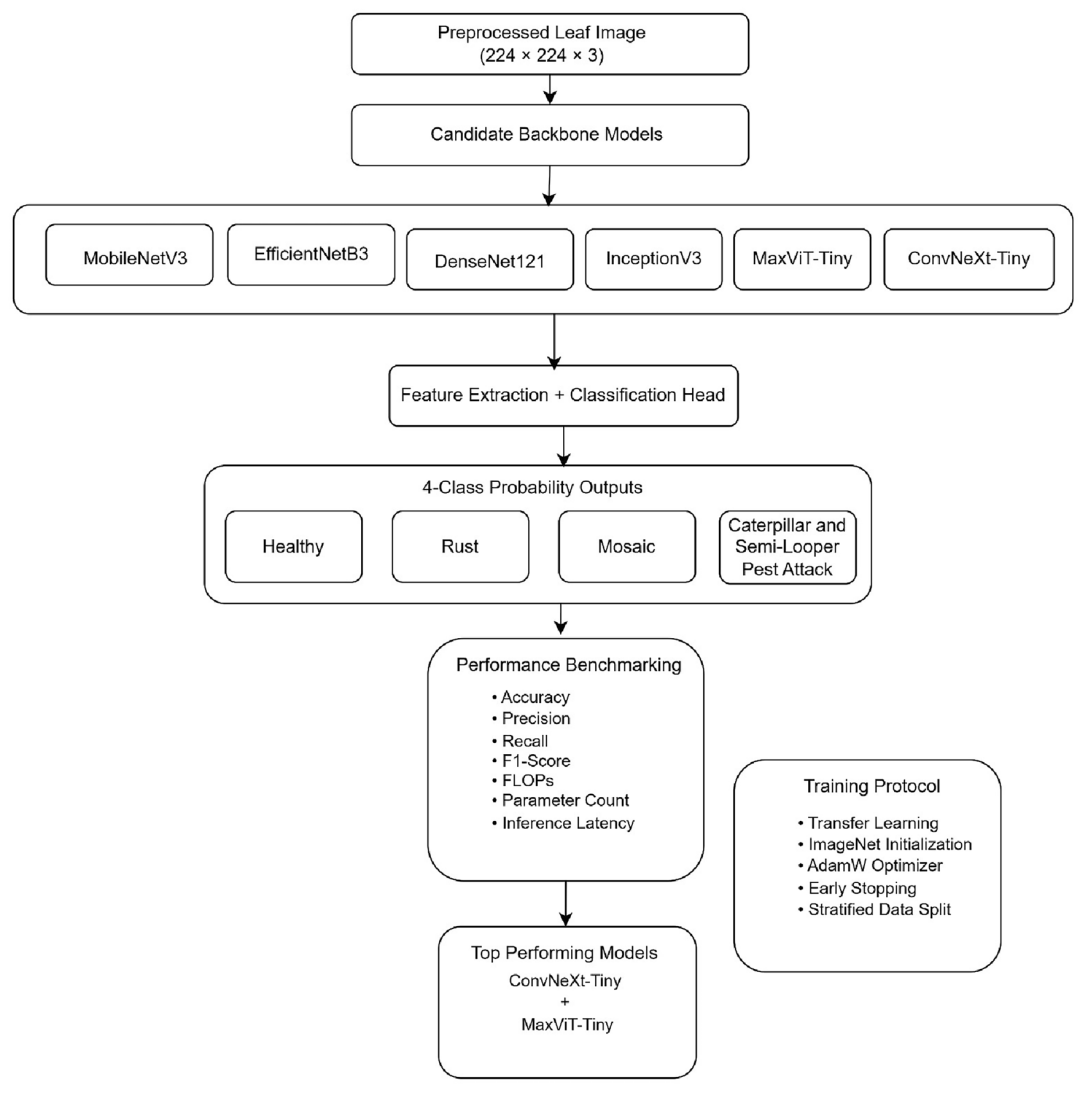
Backbone benchmarking framework showing parallel evaluation of six candidate architectures on preprocessed 224*×*224 leaf imagery. All models share identical training configuration and produce 4-class softmax probability outputs evaluated across accuracy, precision, recall, F1-score, FLOPs, parameter count, and inference latency. ConvNeXt-Tiny and MaxViT-Tiny were selected as top-performing models for ensemble construction.

To establish a strong baseline, six architectures were evaluated, namely, MobileNetV3-Small, DenseNet121, InceptionNet, EfficientNet-B3, ConvNeXt-Tiny, MaxViT-Tiny. Figure 6 illustrates the backbone benchmarking protocol including parallel evaluation of all six architectures, performance metrics, and training configuration.

Lightweight architectures such as MobileNet have been very commonly used in plant disease detection due to its feasibility in deployment^22^. Hybrid CNN-transformer models such as ConvNeXt and trasnformer-based architectures have demontrated significant improved performance in complex field environments^29,31,32^.

We have trained all the models using cross-entropy loss:

$$\mathcal {L}_{CE} = - \sum _{c=1}^{C} y_c \log (\hat{y}_c)$$

Finally, to ensure robust feature representation, we selected two state-of-the-art backbones which was based on exhaustive preliminary evaluation of eight different architectures:

ConvNeXt-Tiny (CNN Branch) : This model represents the evolution of standard convolutions and provides strong inductive bias for local spatial features, for example the specific textural irregularities of Pest Attack (holes) and Rust (pustules).

MaxViT-Tiny (Transformer Branch): MaxViT (Multi-Axis Vision Transformer) utilizes a unique grid and block attention mechanism that allows the model to capture the global dependencies as well as long-range spatial relationships which are critical for identifying broader patterns like Mosaic virus yellowing across a leaf or canopy.

### Hybrid fusion strategies

The ensemble strategy combines the class probability outputs of ConvNeXt-Tiny and MaxViT-Tiny, the two highest-performing backbones identified through systematic benchmarking. Rather than combining all six evaluated backbones, the ensemble is deliberately restricted to these two architectures for two reasons. First, including lower-performing models introduces noise into the combined prediction, as weaker models may amplify shared misclassifications rather than correcting them. Second, ConvNeXt-Tiny and MaxViT-Tiny exhibit complementary feature extraction behaviors where ConvNeXt-Tiny captures fine-grained local textural features through its convolutional inductive bias while MaxViT-Tiny captures global spatial dependencies through its multi-axis attention mechanism making their combination particularly effective for ensemble construction.

**Fig. 7:**
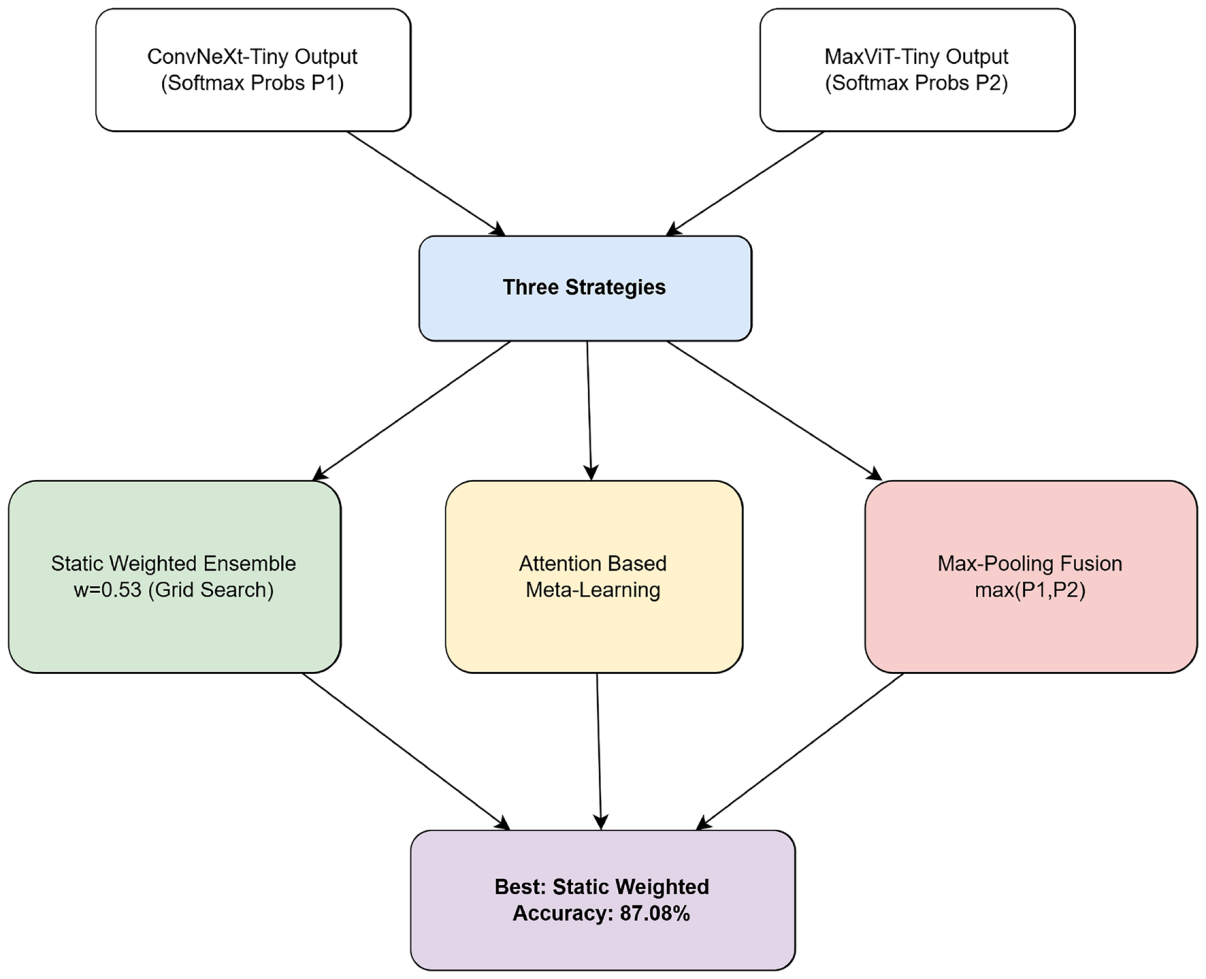
Hybrid fusion strategy framework illustrating three probability-level combination approaches evaluated in this work. ConvNeXt-Tiny and MaxViT-Tiny independently produce softmax probability vectors P1 and P2, which are combined via: (1) Static Weighted Ensemble with optimal weight *w=0.53* identified through grid search, (2) Attention-Based Meta-Learner with dynamic per-image weighting, and (3) Max-Pooling Fusion selecting maximum confidence per class. The static weighted ensemble achieved the best performance with 87.08% accuracy.

Each backbone independently processes the preprocessed input image and produces a softmax probability vector of dimension *C = 4* corresponding to the four disease classes. These probability vectors are combined at the decision level rather than at the feature level, preserving the independently optimized classification boundaries of each backbone. Three probability-level fusion strategies were evaluated to determine the optimal combination mechanism, as illustrated in Fig. 7 and described below.

#### Static weighted ensemble (optimized via grid search)

The first fusion strategy we looked into used a weighted linear combination of the class probability distributions that are generated by the two backbones. To avoid random weight selection, a systematic grid search was conducted over the weight parameter *w ∈ [0,1]* with a step size of 0.01.

$$P_{fused} = w \cdot P_{ConvNeXt} + (1 - w) \cdot P_{MaxViT}$$

where *w ∈ [0, 1]* is the ensemble weight controlling the relative contribution of each backbone. To avoid arbitrary weight selection, a systematic grid search was conducted over the full weight space with a step size of *Δ w = 0.01*, evaluating ensemble validation accuracy at each weight value. The optimal weight *w = 0.53* was identified, indicating near-equal but not identical contributions from each backbone - ConvNeXt-Tiny contributing slightly more, consistent with its stronger local texture discrimination capability for fine-grained lesion features. The final predicted class is determined as:

$$\hat{y} = \arg \max _{c} \; P_{fused}(c)$$

No additional training is required for the static weighted ensemble beyond individual backbone training, as it operates purely through post-hoc probability combination at inference time.

#### Dynamic attention-based meta-learner

The second fusion strategy employs a Multi-Head Attention-based gating mechanism to enable image-specific adaptive weighting. Unlike the static ensemble which assigns fixed weights regardless of input content, this meta-learner computes dynamic weights for each input sample based on the confidence distributions of both backbones. The gating module projects the logits from both backbones into a shared latent representation and applies self-attention to the concatenated logits, allowing the meta-learner to estimate a dynamic weight vector per image and effectively select the most confident expert for each specific input.

Given the moderate size of the leaf dataset, strong regularization was applied to prevent meta-learner overfitting: L2 weight decay ($10^{-3}$) and Dropout (0.5) were incorporated within the gating network, and backbone weights were frozen during meta-learner training. Despite its theoretical adaptability, the dynamic attention fusion did not outperform the optimized static weighted ensemble, suggesting that the dataset size limits effective learning of input-specific weighting and that the additional variance introduced by the learned gating mechanism outweighs its potential benefits under moderate data conditions.

#### Max-pooling fusion

For having a non-linear baseline, we implemented a max-pooling fusion strategy:

$$P_{fused}(c) = \max \left( P_{ConvNeXt}(c), P_{MaxViT}(c)\right)$$

This approach prioritizes the strongest per-class prediction from either backbone, operating on the assumption that at least one model will be highly confident for the correct class. While conceptually appealing as a confidence-based aggregation mechanism, this strategy also did not outperform static weighted averaging. This outcome indicates limited complementary error patterns between the two backbones. Since both architectures exhibited similar confidence distributions and overlapping misclassifications on difficult samples, selecting the maximum probability amplified shared errors rather than correcting them. This finding suggests that max-pooling fusion is most effective when constituent models have more divergent confidence profiles than observed here.

### Generalizability and regularization strategies

Given the moderate size of the MH-SoyaHealthVision dataset and its four disease categories, several strategies were specifically adopted to improve generalization and reduce overfitting. First, all backbone models were initialized using ImageNet pre-trained weights, providing strong low-level feature representation and reducing the dependence on large task-specific datasets. Second, labels smoothing (0.1) was incorporated during training to reduce overconfident predictions and improve the model calibration under limited data conditions. Third, cosine annealing learning rate scheduling was used to encourage smoother convergence and avoid sharp minima that often generalize poorly. Fourth, AdamW optimization with weight decay (0.05) introduced additional regularization to further limit overfitting. Fifth, stratified dataset splitting ensured balanced class representation across training, validation, and test sets despite the presence of class imbalance. Finally, supervised contrastive learning also served as a form of structural regularization by encouraging better organization of the embedding space beyond just a simple decision boundary optimization, which can improve generalization in limited data scenarios.

### Cross-scale UAV transfer

The practical motivation for cross-scale transfer is not primarily annotation cost and we acknowledge that image-level labels for UAV imagery are no harder to obtain than leaf-level labels. Rather, the motivation is data collection asymmetry: ground-level leaf photography requires only a smartphone and field access, whereas UAV data collection requires specialized drone equipment, regulatory flight permissions, and coordinated field campaigns. Leaf-level disease datasets are therefore substantially more abundant in existing literature and more accessible to resource-constrained research groups. The central practical question motivating this progressive evaluation is therefore: given this data collection asymmetry, how effectively can leaf-level representations transfer to UAV-scale inference, and what alignment strategies best minimize the resulting domain gap?

To establish a quantitative lower bound on cross-scale transfer performance, the leaf-trained ensemble was directly evaluated on UAV imagery without any domain adaptation. This zero-shot assessment serves as a baseline that motivates the subsequent fine-tuning and supervised contrastive learning stages described below.

#### Fine-tuning for domain adaptation

Fine-tuning for UAV domain adaptation was performed by initializing the UAV training process with weights learned from leaf-level classification. The transfer of knowledge operates through the hierarchical feature representations encoded across the network layers. Early convolutional layers in both ConvNeXt-Tiny and MaxViT-Tiny encode domain-agnostic low-level features such as edge patterns, color gradients, texture primitives, and vein structures that are physically present in both leaf-level and UAV-scale imagery regardless of sensing scale. These representations are transferable across domains and were therefore preserved by applying a reduced learning rate of $\frac{LR}{10}$ to the backbone layers during UAV fine-tuning, preventing catastrophic forgetting of generalizable low-level features. - In contrast, the deeper layers of the network encode high-level semantic representations that are domain-specific and are optimized for the spatial framing, background distribution, and lesion scale characteristic of leaf-level imagery. These layers require adaptation to UAV-specific characteristics such as canopy-level occlusion, reduced lesion visibility, and increased background clutter. Accordingly, the classification head was updated at the full learning rate to allow rapid adaptation of task-specific decision boundaries to the UAV domain. This differential learning rate strategy preserves transferable low-level knowledge while enabling high-level semantic adaptation, consistent with transfer learning principles established in cross-domain agricultural vision research^25,29^.

### Supervised contrastive learning

To further improve cross-scale feature alignment, supervised contrastive learning was implemented with three specific design decisions tailored to the multi-scale agricultural domain.

First, the positive pair sampling strategy was specifically adapted for cross-scale alignment: images sharing the same disease class label are treated as valid positive pairs regardless of whether they originate from the leaf or UAV domain. This explicitly encourages the network to learn class-consistent representations that are invariant to sensing scale and viewpoint differences, rather than simply clustering within-domain samples of the same class. Unlike standard supervised contrastive learning applied within a single domain, this cross-domain positive pairing directly targets the leaf-to-UAV domain gap.

Second, joint mini-batch construction was designed to ensure balanced sampling of both leaf and UAV images within each training batch. This prevents the contrastive loss from being dominated by within-domain pairs, which would reduce the cross-scale alignment effect and cause the model to learn domain-specific rather than domain-generalizable representations.

Third, the contrastive loss is combined with standard cross-entropy loss using a weighted formulation that preserves classification boundary optimization while simultaneously enforcing embedding space structure:

$$\mathcal {L} = \mathcal {L}_{CE} + \lambda \mathcal {L}_{SupCon}$$

where *λ = 0.5* was selected to balance classification performance and embedding alignment. The supervised contrastive loss is defined as:

$$\mathcal {L}_{SupCon} = \sum _{i=1}^{N} \frac{-1}{|P(i)|} \sum _{p \in P(i)} \log \frac{ \exp (z_i \cdot z_p / \tau ) }{ \sum _{a \ne i} \exp (z_i \cdot z_a / \tau ) }$$

where $z_i$ denotes the normalized embedding of the anchor sample, *P*(*i*) denotes the set of positive samples sharing the same class label as anchor *i* across both leaf and UAV domains, and *τ = 0.07* is the temperature parameter controlling the sharpness of the distribution.

These three design decisions collectively adapt the contrastive framework specifically for cross-scale agricultural feature alignment. By jointly sampling leaf and UAV images within the same mini-batch, the model learns to minimize intra-class domain discrepancy while maximizing inter-class separation, thereby improving cross-scale generalization beyond what fine-tuning alone achieves^36^.

### Feature embedding analysis

We extract the feature embeddings from the penultimate layer and apply PCA followed by t-SNE for visualization. We then compute the silhouette scores to quantify the cluster quality:

$$S = \frac{b - a}{\max (a,b)}$$

where *a* denotes intra-cluster distance and *b* denotes nearest-cluster distance.

### Interpretability via Grad-CAM

To enhance model transparency we made use of Grad-CAM^37^. Activation maps were computed based on:

$$\alpha _k = \frac{1}{Z} \sum _i \sum _j \frac{\partial y^c}{\partial A_{ij}^k}$$

$$L^{GradCAM} = \text {ReLU}\left( \sum _k \alpha _k A^k \right)$$

Heatmaps confirmed that the model focused on the biologically meaningful lesion regions rather than focusing on background details.

### Summary of methodological contributions

The proposed methodology contributes:

- Systematic backbone benchmarking under multi-scale conditions.
- Optimized weighted ensemble for improved leaf-level accuracy.
- Quantitative cross-domain evaluation via zero-shot transfer.
- Contrastive learning for feature alignment across sensing scales.
- Embedding-level validation using silhouette metrics.

## Experimental setup

This section emphasizes on the complete implementation details and training configuration which we use to evaluate the mutli-scale soybean health assessment framework and the objective is to ensure full reproducibility and fair comparision across all backbone architectures, fusion strategies, and cross-scale transfer experiments.

### Hardware and software environment

All the experiments were implemented with the help of Pytorch (v2.x) with CUDA acceleration and training and evaluation was conducted on an NVIDIA Tesla T4 GPU with 16GB VRAM. Mixed precision training was also employed using automatic mixed precision (AMP) so that we can improve computational efficiency as well as reduce memory consumption. Finally, to ensure reproducibility we conducted all experiments using a fixed random seed (seed = 42).

### Dataset splitting protocol

A stratified splitting strategy of 70:15:15 was applied to both leaf and UAV datasets independently to preserve class distribution consistency across training, validation, and test sets. The resulting split sizes are summarized in Table 5.

**Table 5:** Dataset split sizes for leaf and UAV datasets.

Dataset	Total	Train (70%)	Val (15%)	Test (15%)
Leaf	2835	1984	426	425
UAV	2845	1991	427	427

All reported performance metrics correspond to the held-out test sets unless otherwise specified. The test sets were not used at any point during model development, hyperparameter selection, or ensemble weight optimization, ensuring unbiased final evaluation.

Regarding partitioning granularity, the MH-SoyaHealthVision dataset was partitioned at the image level using stratified random sampling. The original dataset documentation does not provide plant-level, plot-level, or field-level identifiers that would enable spatial partitioning. We acknowledge that image-level splitting may not guarantee complete spatial independence between individual plants or field plots, as this depends on the collection protocol of the original dataset. This is explicitly noted as a limitation, and we recommend that future agricultural dataset releases standardize spatial metadata reporting to enable field-level partitioning.

For cross-scale evaluation the following protocol was applied.

- Leaf models were trained exclusively on the leaf training set.
- Zero-shot evaluation was performed directly on the UAV test set without any retraining.
- Fine-tuning and supervised contrastive learning were performed using the UAV training set, with model selection based on UAV validation set performance.

### Input configuration and normalization

All the input images were resized to *224 × 224* pixels after square padding to preserve the aspect ratio and normalization was performed using ImageNet statistics:

$$\mu = (0.485, 0.456, 0.406), \quad \sigma = (0.229, 0.224, 0.225)$$

Mini-batch size was set to 16 for all the experiments.

It is important to acknowledge the resolution trade-off introduced by downsampling high-resolution UAV imagery to 224*×*224 pixels for backbone compatibility. The DJI Mini 4 Pro captures UAV imagery at 3840*×*2160 pixels, representing a linear downsampling factor of approximately 17*×* relative to the network input resolution. At UAV flight altitudes of 12–25 meters, individual disease lesions may occupy only a small spatial region within the full-resolution image, and aggressive downsampling risks compressing fine-grained lesion detail that would otherwise be diagnostically informative.

To address this concern, two alternative strategies were empirically evaluated. First, higher input resolutions were tested but could not be sustained on the available NVIDIA Tesla T4 GPU hardware due to memory constraints, making 224*×*224 the maximum feasible resolution within the experimental environment. Second, a patch-based inference strategy was evaluated, in which full-resolution UAV images were divided into overlapping spatial tiles before classification. However, this approach yielded degraded performance compared to whole-image resizing, likely because patch-level cropping disrupts the canopy-scale contextual features such as spatial lesion distribution and inter-plant variation that are critical for UAV-scale disease recognition. Based on these empirical findings, 224*×*224 whole-image resizing with aspect-ratio-preserving square padding was adopted as the optimal feasible configuration. To mitigate residual information loss, supervised contrastive learning encourages the network to learn class-discriminative representations from higher-level structural and contextual features that remain discernible at this resolution even when fine-grained lesion textures are partially compressed.

### Backbone training configuration

Six backbone architectures were evaluated during leaf-level benchmarking: MobileNetV3-Small, DenseNet121, InceptionNet, EfficientNet-B3, ConvNeXt-Tiny, and MaxViT-Tiny. All networks were initialized with ImageNet pretrained weights. A unified training configuration was applied across all backbones to ensure fair and reproducible comparison, as summarized in Table 6.

**Table 6:** Hyperparameter configuration for backbone training.

Hyperparameter	Value
Optimizer	AdamW
Initial learning rate	$3 \times 10^{-4}$
Weight decay	0.05
Batch size	16
Training epochs	30
Loss function	Cross-entropy with label smoothing
Label smoothing	0.1
Learning rate schedule	Cosine annealing
Input resolution	224*×*224 pixels
Precision	Mixed (AMP)
Random seed	42

The cosine annealing learning rate schedule was applied as:

$$\eta _t = \eta _{min} + \frac{1}{2}(\eta _{max} - \eta _{min}) \left( 1 + \cos \left( \frac{t}{T}\pi \right) \right)$$

where *T* denotes the total number of training steps. The best-performing model checkpoint was selected based on maximum validation accuracy.

Regarding hyperparameter optimization: the learning rate of $3 \times 10^{-4}$, weight decay of 0.05, and batch size of 16 were selected based on established best practices for ImageNet-pretrained fine-tuning in agricultural vision tasks^29,35^. Label smoothing of 0.1 was selected as a standard regularization value for multi-class classification under moderate dataset sizes. The number of training epochs was set to 30 based on preliminary validation convergence analysis, after which no further improvement in validation accuracy was observed across all backbone architectures. A systematic hyperparameter search was not conducted for all parameters given computational constraints; however, the cosine annealing schedule and AdamW optimizer were selected for their well-documented stability and generalization properties in transformer-based and CNN-based vision models.

#### Optimization settings

We made use of the following optimization configuration for all backbone training:

- Optimizer: AdamW
- Initial Learning Rate: $3 \times 10^{-4}$
- Weight Decay: 0.05
- Epochs: 30
- Loss Function: Cross-Entropy Loss with label smoothing (0.1)

We also applied a cosine annealing learning rate schedular:

$$\eta _t = \eta _{min} + \frac{1}{2}(\eta _{max} - \eta _{min}) \left( 1 + \cos \left( \frac{t}{T}\pi \right) \right)$$

where *T* denotes the total number of training steps. The best-performing model was selected based on the maximum validation accuracy and the corresponding checkpoints were saved.

### Hybrid fusion configuration

ConvNeXt-Tiny and MaxViT-Tiny were selected for ensemble experiments.

#### Static weighted ensemble

A grid search was conducted over the weight parameter:

$$w \in [0,1], \quad \Delta w = 0.01$$

The optimal weight was selected based on validation accuracy.

#### Dynamic attention-based meta-learner

The gating mechanism consisted of:

- Linear projection layers
- Multi-head self-attention
- Dropout (0.5)
- L2 weight decay ($$10^{-3}$$)

Backbone weights were frozen during meta-learner training to prevent overfitting.

#### Max-pooling fusion

Element-wise maximum fusion was implemented across class probability vectors.

### Cross-scale transfer protocol

#### Zero-shot transfer

We first directly evaluated the leaf-trained models on UAV test data without retraining to quantify the domain shift.

#### Fine-tuning strategy

For UAV domain adaptation, the following layer-wise transfer strategy was employed:

- *Backbone layers (frozen at reduced learning rate)* Early and intermediate convolutional layers were updated at $\frac{LR}{10}$ to preserve low-level transferable features including edge patterns, color gradients, texture primitives, and vein structures that are domain-agnostic across leaf and UAV scales.
- *Classification head (full learning rate)* The final classification layers were updated at the full learning rate ($3 \times 10^{-4}$) to enable rapid adaptation of high-level semantic representations to UAV-specific spatial distributions, background complexity, and canopy-scale disease patterns.
- *Training duration* 30 epochs with cosine annealing learning rate scheduling.
- *Initialization* UAV training initialized with leaf-pretrained weights from the best-performing MaxViT-Tiny checkpoint selected based on leaf validation accuracy.

This differential learning rate strategy is grounded in the hierarchical nature of deep network representations: lower layers encode transferable domain-agnostic features while upper layers encode domain-specific semantic abstractions that require adaptation to the target domain.

#### Supervised contrastive learning

For feature alignment we performed supervised contrastive learning using joint batches of both leaf and UAV images.

Training parameters included:

- Projection head dimension: 128
- Temperature parameter: *τ = 0.07*
- Contrastive loss weight: *λ = 0.5*

The total loss function was defined as:

$$\mathcal {L} = \mathcal {L}{CE} + \lambda \mathcal {L}{SupCon}$$

### Feature embedding analysis

We extracted the feature embeddings from the penultimate layer of the network.

Dimensionality reduction was performed using:

- Principal Component Analysis (PCA) to 50 dimensions
- t-SNE projection to 2 dimensions (perplexity = 30, random state = 42)

Silhouette scores were computed to quantify:

- Inter-class separability
- Domain separability (Leaf vs UAV)

### Evaluation metrics

Model performance was evaluated using:

- Overall Accuracy
- Precision
- Recall
- F1-score
- Confusion Matrix
- Silhouette Score

Overall accuracy is defined as:

$$Accuracy = \frac{TP + TN}{TP + TN + FP + FN}$$

We reported the macro-averaged F1-score to mitigate the issue of class imbalance and the final model selection was based on validation accuracy, convergence stability and cross-domain generalization performance.

## Results and analysis

This section presents a complete evaluation of our proposed multi-scale soybean health assessment framework. The results are structured into four major parts: (1) leaf-level backbone benchmarking, (2) hybrid fusion analysis of the best models, (3) cross-scale UAV transfer evaluation, and (4) feature alignment and interpretability analysis

### Leaf-level backbone benchmarking

For establishing a strong baseline we evaluated six different backbone architectures on the leaf dataset. The quantitative results are summarized in Table 7.

**Table 7:** Leaf-level backbone benchmarking results.

Model	Accuracy	Precision	Recall	F1-score	Params	FLOPs	Inference
	(%)	(%)	(%)	(%)	(M)	(G)	(ms)
MobileNetV3-S	74.44	77.75	78.00	77.75	1.5	0.06	5.45
InceptionNet	75.28	79.00	79.25	78.75	25.1	2.85	11.61
DenseNet121	77.53	80.50	81.00	80.50	7.0	2.90	15.12
EfficientNetB3	78.37	81.75	81.50	81.75	10.7	1.02	13.15
ConvNeXt-Tiny	83.15	85.25	85.75	85.25	27.8	4.46	5.92
MaxViT-Tiny	84.83	87.00	87.25	87.25	30.4	5.46	28.88

Based on the benchmarking results summarized in Table 7, ConvNeXt-Tiny and MaxViT-Tiny were selected as the final backbones for ensemble construction. While MobileNetV3-Small offers the smallest footprint at 1.5M parameters and the fastest inference at 5.45ms, its classification accuracy of 74.44% is insufficient for reliable disease monitoring. EfficientNetB3 provides a competitive accuracy-efficiency trade-off at 10.7M parameters and 1.02 GFLOPs, but its accuracy of 78.37% still falls short of the top performers. ConvNeXt-Tiny achieves 83.15% accuracy with a notably fast inference time of 5.92ms despite its 27.8M parameters, making it the most computationally efficient among the high-performing models. MaxViT-Tiny achieves the highest individual accuracy of 84.83% at 30.4M parameters and 5.46 GFLOPs, with an inference time of 28.88ms that remains acceptable for non-real-time agricultural monitoring applications. Critically, ConvNeXt-Tiny and MaxViT-Tiny exhibit complementary feature extraction behaviors, ConvNeXt-Tiny excels at capturing fine-grained local textural features such as rust pustules and pest-induced perforations through its strong convolutional inductive bias, while MaxViT-Tiny captures global spatial dependencies critical for identifying broader patterns such as mosaic virus yellowing through its multi-axis attention mechanism. This architectural complementarity, combined with their superior classification performance, justifies their selection for ensemble construction despite their higher computational cost relative to lightweight alternatives.

#### Hybrid fusion strategy evaluation

To investigate different or non-overlapping feature synergy, three fusion strategies were evaluated: static weighted ensemble, attention-based gating, and max-pooling fusion. The comparative results are summarized in Table 8.

**Table 8:** Hybrid fusion strategy comparison.

Fusion Method	Accuracy	F1-score	Params (M)	FLOPs (G)	Inference (ms)
ConvNeXt-Tiny Only	83.15	85.25	27.8	4.46	5.92
MaxViT-Tiny Only	84.83	87.25	30.4	5.46	28.88
Static weighted ensemble	**87.08**	**88.99**	**58.2**	**9.92**	**31.54**
Attention-based meta-learner	86.52	88.53	58.2	9.92	31.85
Max-pooling fusion	86.24	87.94	58.2	9.92	31.74

All three fusion strategies share identical backbone computational cost (58.2M parameters, 9.92 GFLOPs). Inference time differences between fusion methods are within measurement noise as the fusion operation contributes negligible overhead relative to the backbone forward passes.

Significant values are in [bold].

**Fig. 8:**
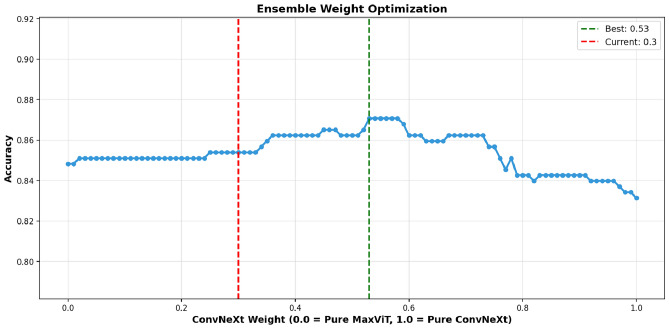
Grid search optimization curve for weighted ensemble.

We observed that the static weighted ensemble achieved the best overall performance with an optimal weight of *w = 0.53* identified through grid search. All three fusion strategies share identical backbone computational cost since they process input through the same ConvNeXt-Tiny and MaxViT-Tiny backbones, with fusion operations contributing negligible additional overhead. The grid search optimization curve is illustrated in Fig. 8.

We can observe that interestingly the attention-based meta-learner did not outperform static weighted averaging, which suggests that dynamic gating maybe constrained by dataset size and may introduce additional variance under limited amount of training data.

Similarly, even Max Pooling did not improve performance which indicates limited complementary error patterns between the base models and since both architectures exhibited similar confidence distributions and overlapping misclassifications, selecting the maximum probability instead amplified shared errors rather than correcting them.

#### Detailed performance of the best static weighted ensemble

Since the optimized static weighted ensemble achieved the highest leaf-level performance we have provided a detailed class-wise analysis in this subsection.

Table 9 summarizes the per-class precision, recall, F1-score, and support values for the best ensemble configuration.

**Table 9:** Class-wise performance of best static weighted ensemble (leaf dataset).

Class	Precision	Recall	F1-score	Support
Healthy	100%	100%	100%	32
Mosaic	84.35%	90.65%	87.39%	107
Caterpillar and semi-looper pest attack	81.40%	79.55%	80.46%	88
Rust	90.24%	86.05%	88.10%	129

**Fig. 9:**
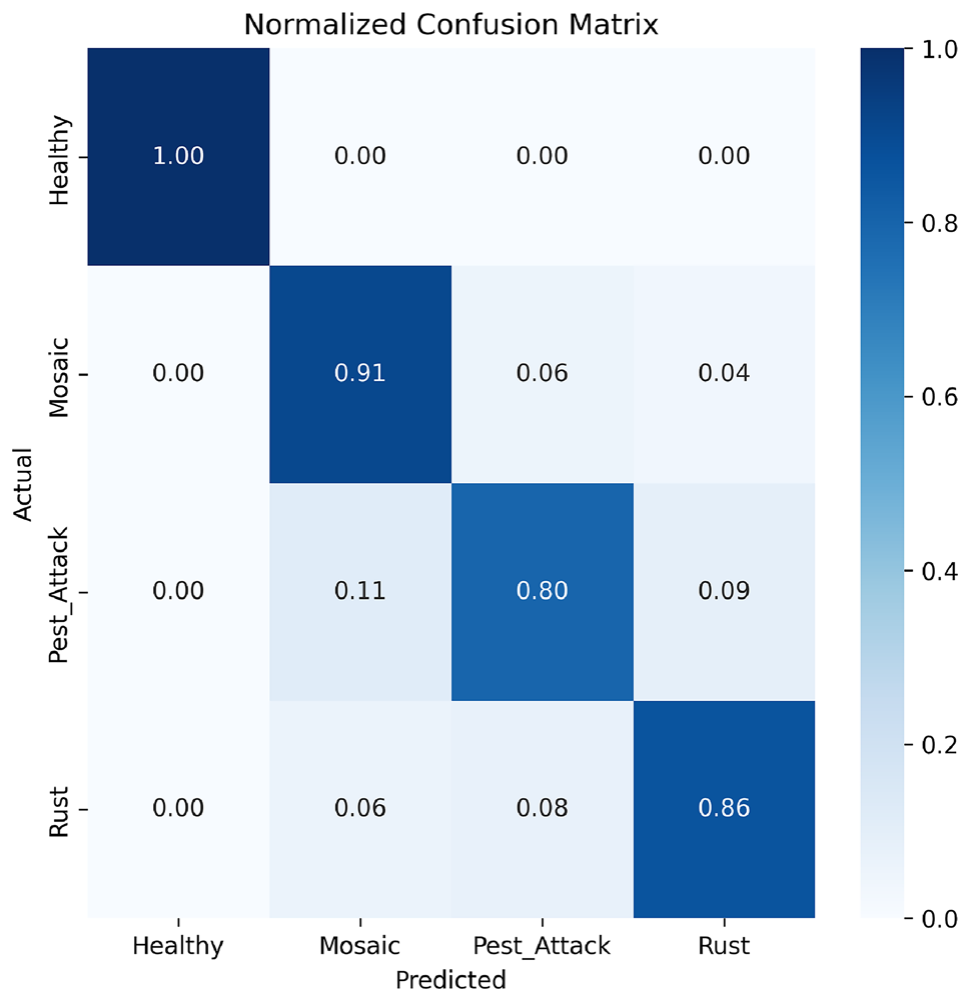
Normalized confusion matrix of the best static weighted ensemble on the leaf test set.

To further analyze mis-classification behaviour we provide the normalized confusion matrix of the best ensemble as shown in Fig. 9.

The normalized matrix gives a clearer interpretation of the class-level prediction distribution by eliminating the influence of class-level imbalance. As we can observe most samples are concentrated along the diagonal which indicates strong intra-class consistency. Minor confusion can also be observed between visually similar disease categories particularly as seen between Pest Attack and Rust which share overlapping lesion patterns under certain illumination conditions.

### Grad-CAM interpretability analysis

**Fig. 10:**
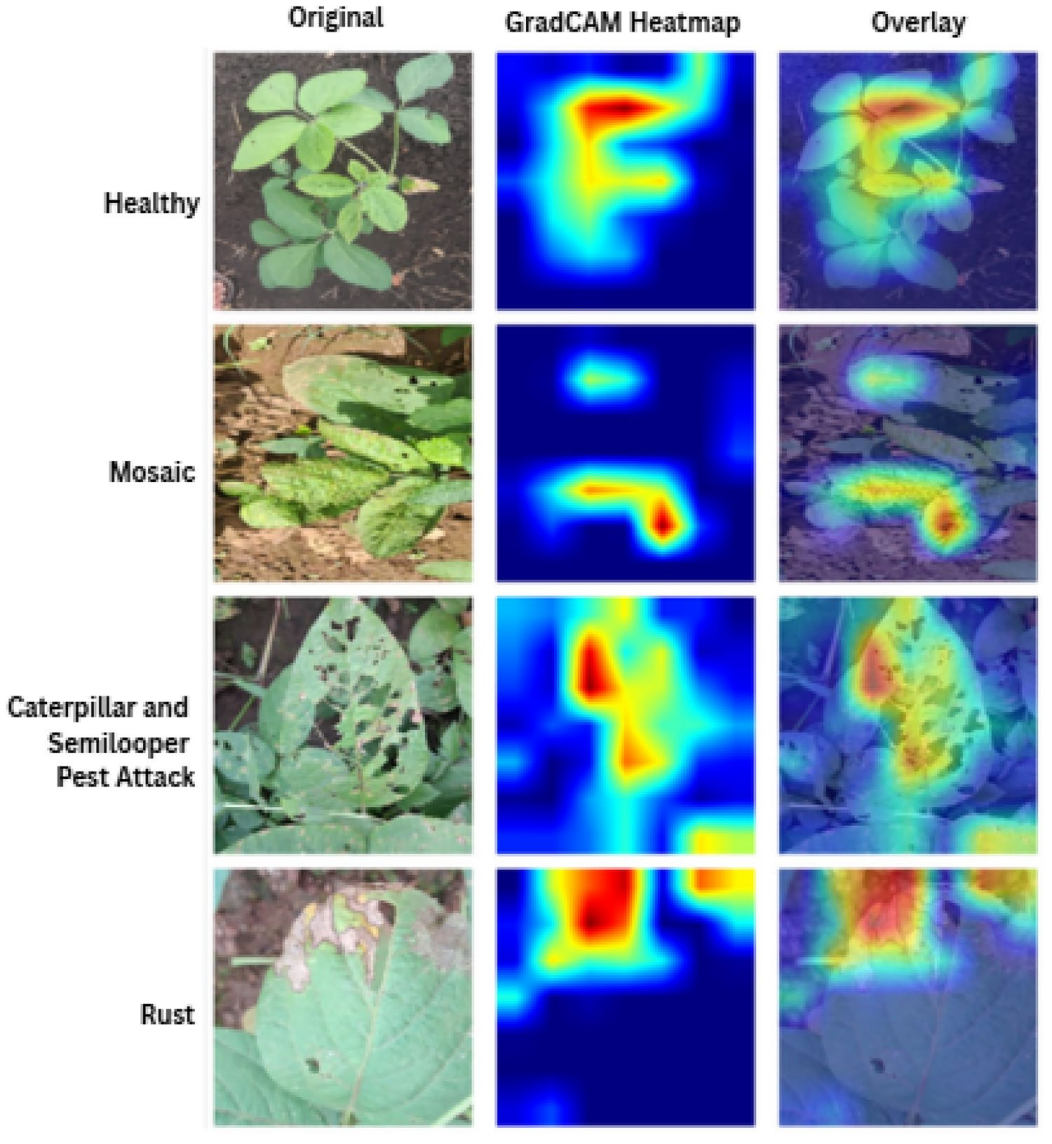
Grad-CAM visualizations for representative leaf samples across four disease classes.

To validate the model attention behaviour, we used Grad-CAM to generate visualizations for representative leaf samples across all four classes. The activation maps are shown in Fig. 10

The activation maps clearly demonstrate that the ensemble model consistently focuses on symptomatic regions such as chlorotic discoloration, necrotic lesions, and pest-induced damage patterns. In case of healthy samples, the attention is distributed across intact leaf structures whereas if we look at diseased samples it shows concentrated activations over affected regions and hence these results confirm that the model learns biologically meaningful features rather than background details.

**Table 10:** Quantitative Grad-CAM analysis: foreground activation ratio (FAR) per disease class.

Class	Mean FAR	Std	N images
Healthy	79.8%	19.5%	32
Mosaic	91.7%	16.4%	107
Pest Attack	90.6%	16.7%	88
Rust	87.5%	18.8%	129
Overall	88.8%	18.0%	356

To provide quantitative grounding for the interpretability analysis, a Foreground Activation Ratio (FAR) metric was computed across all 356 leaf-level test images. FAR measures the proportion of total Grad-CAM activation mass concentrated within leaf and disease-relevant regions versus background areas, using HSV color-based segmentation to identify the foreground region of interest. As reported in Table 10, the ensemble model directs an average of 88.8% (±18.0%) of its activation mass toward leaf foreground regions across all disease classes. Mosaic and Pest Attack classes achieved the highest foreground activation ratios of 91.7% and 90.6% respectively, consistent with their visually distinctive symptom patterns that provide strong localized activation signals. The Healthy class exhibited a slightly lower ratio of 79.8%, which is expected as healthy leaves lack concentrated disease lesions and activation is more uniformly distributed across the intact leaf surface. These results confirm that the model consistently directs attention toward biologically relevant image regions rather than background artifacts, supporting the validity of the learned representations. We acknowledge that IoU against expert-annotated lesion masks would constitute a more rigorous localization metric and identify this as a future work direction pending availability of fine-grained lesion annotations for the MH-SoyaHealthVision dataset.

### Cross-scale UAV transfer evaluation

To assess and analyze domain generalization we evaluated three cross-scale evaluation strategies:

- Zero-shot transfer
- Fine-tuning
- Supervised contrastive learning

The results are presented in Table 11.

**Table 11:** Cross-scale UAV transfer results across five independent random seeds.

Method	Run 1	Run 2	Run 3	Run 4	Run 5	Mean	Std	F1-score
Zero-shot	–	–	–	–	–	39.72%	–	31.75%
Fine-tuned	96.26%	95.33%	96.73%	95.79%	96.96%	96.21%	0.60%	97.75%
Contrastive	97.20%	98.13%	96.73%	97.20%	96.96%	97.24%	0.48%	98.25%

Zero-shot transfer is a deterministic evaluation requiring no training and is therefore reported as a single run. Fine-tuned and contrastive results are reported across five independent random seeds (seeds: 42, 123, 456, 789, 1024)

The accuracy declined to approximately 40% when we directly evaluated the leaf-trained ensemble on the UAV dataset without any retrain, which resulted in a major drop in performance. This sharp decline highlights the severity of the cross-scale domain shift between ground-level leaf images and aerial UAV imagery. Compared to leaf images, which mainly capture fine-grained lesion textures under relatively controlled framing, UAV images contain more background clutter, reduced lesion visibility, and a significant scale variation. These zero-shot transfer results clearly show that effective multi-scale soybean disease identification cannot be achieved using only single-domain training.

In order to reduce this domain mismatch, fine-tuning was performed by initializing UAV trainings with weights learned from the leaf dataset. Across the five independent random seeds, the fine-tuned model achieved a mean accuracy of 96.21% (±0.60%), suggesting that the early convolutional layers learned transferable low-level features such as edge patterns, vein structures, and lesion textures that remain useful across image scales. Fine-tuning allows the higher-level layers to adapt to UAV-specific spatial characteristics while still preserving the foundational representations that support the effectiveness of transfer learning for cross-domain agricultural image analysis.

Although fine-tuning significantly improved classification accuracy and performance, embedding analysis still showed some remaining domain separation. To further reduce this discrepancy, supervised contrastive learning was introduced to organize the feature space by bringing embeddings from the same class closer together, regardless of whether they originated from leaf or UAV imagery, while simultaneously separating embeddings from different classes. Across five independent random seeds, the contrastive model achieved a mean accuracy of 97.24% (±0.48%), with the lower standard deviation compared to fine-tuning (0.48% vs 0.60%) confirming improved convergence stability across random initializations. It is important to note that with only five experimental runs, statistical tests such as Wilcoxon signed-rank test have a minimum achievable p-value of 0.0625, thus making it mathematically impossible to satisfy the conventional threshold of *p < 0.05* regardless of result consistency. Therefore, the contribution of contrastive learning is better shown through its improved convergence stability and enhanced embedding space organization, as further discussed through silhouette analysis in Section 5.4.

### Feature embedding visualization

**Fig. 11:**
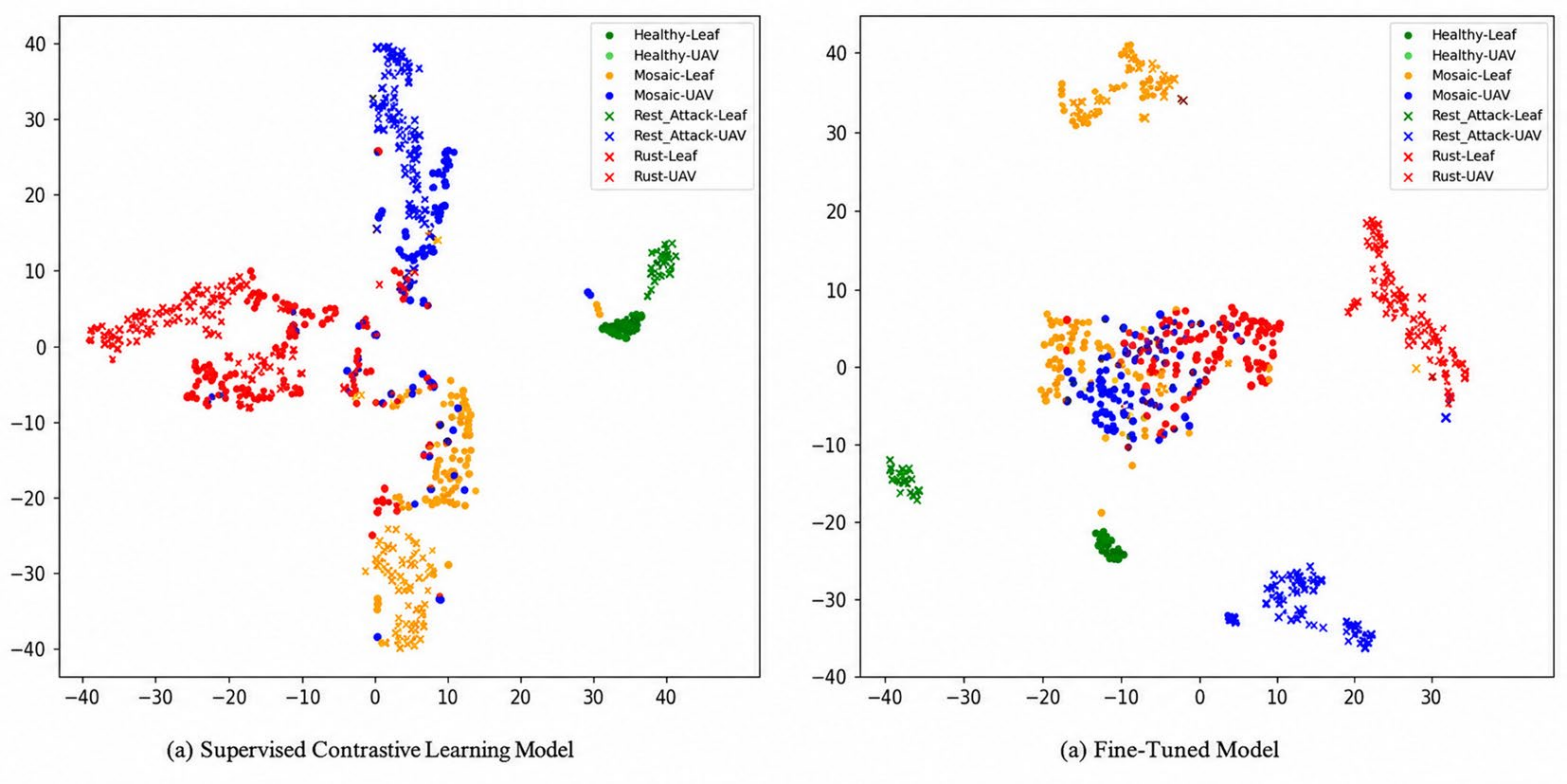
t-SNE visualization of feature embeddings for cross-scale evaluation. (**a**) Supervised contrastive learning model. (**b**) Fine-tuned model. Colours denote disease classes, while markers distinguish leaf and UAV domains.

For analyzing feature alignment PCA followed by t-SNE was applied to the penultimate layer embeddings. Since zero-shot transfer exhibited severe performance degradation we focused the visualizations for the fine-tuned and contrastive models. The visualizations are shown in Fig. 11.

Contrastive learning demonstrated tighter intra-class clustering and improved cross-domain overlap compared to fine-tuning alone as can be seen visually. We also computed silhouette score to quantitatively assess the cluster quality.

**Table 12:** Silhouette analysis for cross-scale feature alignment.

Method	Class Silhouette	Domain Silhouette
Fine-tuned	0.19	0.114
Contrastive	0.59	0.0336

A lower domain silhouette score indicates better cross-domain alignment, whereas a higher class silhouette score indicates stronger class compactness.

As show in Table 12 supervised contrastive learning measurably improves inter-class separability (0.59 vs. 0.19) while simultaneously reducing domain discrepancy (0.0336 vs. 0.114). This reduced domain silhouette score shows stronger mixing between leaf and UAV samples within the same class clusters which confirms improved cross-scale feature alignment.

These results indicate that contrastive learning not only enhances classification accuracy but also structurally reorganizes the embedding space to achieve representations with reduced domain discrepancy. It is important to note that silhouette score improvements serve as a quantitative proxy for embedding space quality rather than a direct measure of agronomic utility. Higher inter-class silhouette scores indicate that the model has learned more discriminative disease representations across scales, which correlates with improved classification performance. We acknowledge that demonstrating direct agronomic utility like for example through integration with farm management decision systems is beyond the scope of this dataset-focused study and identify this as an important direction for future work.

### Class-level performance evaluation of the supervised contrastive model

**Fig. 12:**
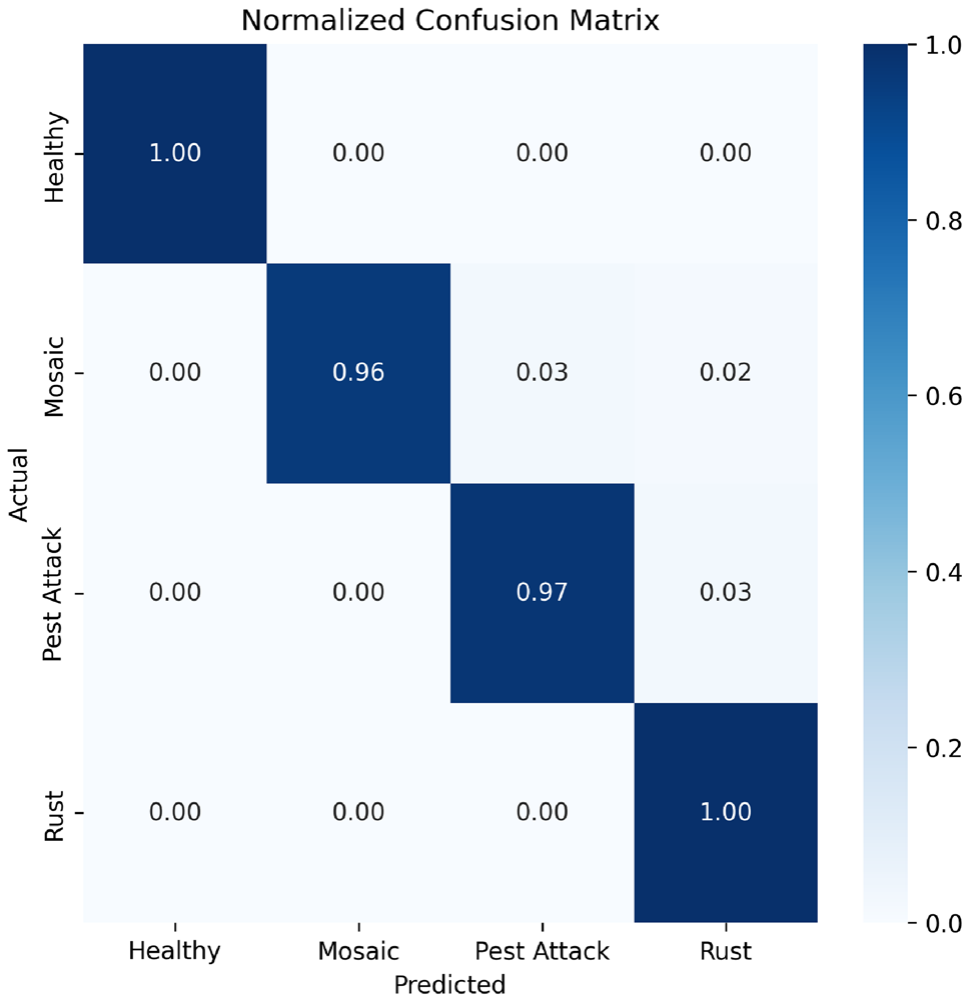
Confusion matrix for supervised contrastive learning model.

The confusion matrix for the best-performing model on the UAV dataset is shown in Fig. 12.

Detailed class-wise performance metrics are summarized in Table 13.

**Table 13:** Class-wise performance metrics (supervised contrastive learning).

Class	Precision	Recall	F1-score
Healthy	100%	100%	100%
Mosaic	100%	95.73%	97.82%
Caterpillar and semi-looper pest attack	97.48%	97.48%	97.48%
Rust	96.77%	100%	98.36%

The results show strong classification performance across all categories, with minor confusion observed between visually similar disease patterns.

### Summary of experimental findings

The experimental evaluation leads to several key conclusions:

- Using Hybrid CNN-transformer ensemble improves leaf-level classification performance.
- Static weighted averaging provides more stable improvements than attention-based fusion under small-to-moderate dataset size.
- Zero-shot transfer exposes substantial cross-scale domain shift between leaf and UAV dataset.
- Fine-tuning measurably mitigates performance degradation.
- Supervised contrastive learning enhances feature geometry, improves inter-class separability, and reduces domain discrepancy.

Collectively, these results validate the proposed pipeline as a practical, scalable, and field-ready solution for real-world disease analysis under constrained annotation settings.

## Discussion

The experimental results demonstrate that reliable soybean disease classification across multiple spatial scales is achievable under real-world agricultural conditions. The proposed framework was evaluated on UAV canopy imagery and ground-level leaf imagery from the MH-SoyaHealthVision dataset^2^ which reflects realistic illumination variation, background clutter, and intra-class variability commonly observed in field environments. Unlike several controlled-environment studies^29,31^, our evaluation considers domain shift between ground-level and aerial imagery, which is a critical challenge in precision agriculture.

A key observation from the experiments is the magnitude of performance degradation under zero-shot transfer from leaf-level training to UAV imagery. While leaf-based classification models achieved strong in-domain performance consistent with prior soybean disease studies^29,33^, direct deployment on UAV data resulted in substantial accuracy reduction. This confirms the presence of significant domain discrepancy between leaf-scale texture patterns and canopy-scale spatial representations, an issue that has been implicitly suggested in cross-domain learning literature^42^ but rarely quantified for soybean disease monitoring.

Fine-tuning of the leaf-pretrained MaxViT backbone substantially improved UAV classification performance, aligning with transfer learning findings reported in plant disease detection research^14,32^. However, the most significant improvement was observed when supervised contrastive learning was introduced to align feature embeddings across scales. The reduction in domain-level silhouette score and simultaneous increase in class-level separability indicate that contrastive alignment promotes domain-invariant yet class-discriminative representations. This supports observations from transformer-based representation learning studies^35,42^, where embedding alignment improves robustness across heterogeneous data distributions.

Another important finding relates to model complexity versus data alignment. Although transformer-based architectures such as ConvTransNet-S^35^ and MobileH-Transformer^18^ have demonstrated improved performance under complex field environments, our results indicate that representation alignment and preprocessing consistency contribute more significantly to cross-scale robustness than architectural depth alone. The ensemble of ConvNext-tiny and MaxViT-tiny achieved superior performance compared to attention-weighted ensembling, suggesting that static weighted averaging may generalize more reliably than learned gating mechanisms under moderate dataset sizes. This observation is consistent with ensemble stability discussions in soybean disease research^33^.

The embedding analysis further strengthens these findings. Principal component analysis and t-SNE visualization reveal improved inter-class separation and reduced domain clustering after contrastive learning. The quantitative silhouette improvements provide objective evidence that feature alignment contributes to biologically meaningful discrimination rather than shortcut learning based on background or illumination artifacts. Similar concerns regarding shortcut learning and environmental bias have been highlighted in agricultural vision literature^34,36^.

Explainability analysis using Grad-CAM further supports the validity of the learned representations. Activation maps consistently focused on disease-specific regions such as lesion patterns, chlorotic patches, and necrotic areas rather than soil background or canopy shadows. This behavior aligns with explainable AI findings in agricultural disease detection^15^, reinforcing that the proposed framework bases predictions on physiologically relevant structures.

Beyond classification performance, the multi-scale alignment framework has broader implications for precision agriculture. UAV-based phenotyping studies typically emphasize yield prediction, chlorophyll estimation, or biomass regression^8,10,13,27^. In contrast, the present framework extends UAV monitoring to categorical disease recognition with validated cross-domain generalization. This integration bridges the gap between leaf-level symptom analysis and canopy-scale surveillance, enabling scalable disease monitoring systems.

Overall, the results confirm that supervised contrastive alignment, systematic backbone benchmarking, and embedding-level validation collectively contribute to a systematic and reproducible soybean disease assessment framework suitable for real-field deployment.

## Limitations

Although the proposed multiscale soybean disease framework showed a strong performance across leaf and UAV imagery, a few limitations were still observed.

First, the supervised contrastive alignment strategy relies on the quality and diversity of class representations across scales. The MH-SoyaHealthVision dataset provides both UAV and leaf imagery , but it also has certain variations in disease severity, crop growth stages, and environmental conditions, which may not be clearly representative. UAV-based agricultural datasets naturally involve variability in altitude, sensor settings, and illumination conditions. These variabilities may introduce moderate domain shifts when models are deployed in geographically cultivated regions.

Second, UAV imagery used in the study is primarily RGB-based. RGB sensing remains widely adopted due to its cost-effectiveness and deployment simplicity, but alongside it also has shown improved sensitivity to biochemical and physiological variations in soybean crops using multispectral and hyperspectral sensing. Combining near-infrared or hyperspectral bands could strengthen the discrimination during the early disease progression stage and in dense canopy situations. Additionally, multispectral systems have also introduced additional hardware requirements and operational considerations.

Third, class imbalance problem still remains as a challenge in agricultural datasets as certain disease categories occur less frequently under natural field conditions. Although class-aware optimization strategies were employed, some variability still remained in the performance. Similar observations regarding dataset imbalance were seen in soybean and broader plant disease detection studies. However, the framework still manages to maintain stable overall performance across categories.

Fourth, we acknowledge that silhouette-based embedding analysis provides a proxy measure of feature alignment quality rather than direct agronomic utility validation. While improved embedding geometry correlates with classification performance gains, its translation to real-world farm management outcomes remains to be demonstrated. Future work should evaluate the proposed framework within an end-to-end crop management decision pipeline to assess practical agronomic impact.

Fifth, the MH-SoyaHealthVision dataset was partitioned at the image level due to the absence of plant-level or field-level spatial identifiers in the dataset documentation. While stratified sampling ensures class distribution consistency across splits, image-level partitioning cannot guarantee complete spatial independence between individual plants across training and test sets. Future work should employ field-level partitioning when dataset spatial metadata permits, to provide stronger guarantees against potential data leakage from spatially proximate samples.

Sixth, a fundamental limitation of the cross-scale transfer framework concerns label semantic correspondence across spatial scales. Leaf-level labels denote disease presence on individual leaves under close-range imaging, while UAV canopy labels denote dominant disease presence across a canopy patch containing multiple plants and leaves in varying health states. While expert annotation ensures label reliability within each domain independently, the semantic correspondence between scales is implicit rather than formally verified through spatial co-registration or quantitative disease severity thresholds. This represents a genuine conceptual limitation: cross-scale contrastive alignment encourages the model to map same-labeled embeddings together across domains, but if label semantics differ substantially across scales, this alignment may partially encode semantic inconsistency rather than genuine disease-specific features. Future work should establish explicit label correspondence protocols, for example by defining quantitative canopy-level disease severity thresholds that correspond to leaf-level symptom presence, to place cross-scale transfer frameworks on a more formally grounded semantic foundation.

Seventh, the UAV annotation workflow in the MH-SoyaHealthVision dataset relies on HSB-based color segmentation and SLIC superpixel generation to compute a disease pixel ratio for canopy patch labeling. However, the original dataset documentation does not specify a precise quantitative threshold for this disease pixel ratio criterion, for example, what minimum proportion of diseased pixels within a canopy patch is required to assign a class label such as “Rust.” This absence of an explicit threshold means that the precise semantic boundary of UAV canopy labels cannot be fully characterized, which has direct implications for cross-scale label correspondence. Future dataset collection efforts should standardize and report quantitative class criteria thresholds to enable more rigorous cross-scale validation.

These limitations depict broader challenges in real-world agricultural AI systems and provide opportunities for future enhancement for more scalable, domain-invariant crop health monitoring frameworks.

## Conclusion and future work

This study showed a unified multi-scale framework for soybean crop health assessment by combining leaf-level imagery with UAV-based canopy observations using the MH-SoyaHealthVision dataset. Multiple deep learning backbones were systematically evaluated for leaf-level classification and then went into cross-scale generalization under realistic field conditions.

Results show that although leaf-level models achieve strong in-domain performance, consistent with previous soybean disease studies, direct zero-shot transfer to UAV imagery leads to performance degradation due to domain shifts. Fine-tuning improves results measurably, and supervised contrastive learning further enhances classification accuracy and cross-scale feature alignment. Silhouette analysis confirms improved inter-class separability after alignment, supporting the development of representations with reduced cross-scale domain discrepancy.

Backbone benchmarking and static weighted ensembling provide stable gains without requiring complex adaptive weighting strategies. Grad-CAM visualizations generate biologically meaningful activation regions with heatmaps which aligns with explainable AI findings in agricultural research, and reinforcing model reliability.

Compared to prior UAV-based soybean studies focused mainly on regression tasks such as yield or biomass estimation and this work extends aerial monitoring to categorical disease classification with validated cross-scale robustness. The proposed framework offers a systematic and interpretable approach for real-field soybean disease assessment.

Future work may include multispectral sensing for improved discrimination, explore semi-supervised or self-training strategies under limited annotations, and explore temporal UAV modeling to capture disease progression dynamics. Efficient transformer variants and model compression techniques could further support edge deployment. Additionally, cross-dataset validation which is training on leaf imagery from one independently collected dataset and evaluating UAV transfer on a separately acquired aerial dataset from a different geographic region represents an important direction to assess the true generalizability of the proposed framework under realistic deployment conditions beyond the single-dataset setting evaluated here. Furthermore, the current framework covers four soybean health conditions reflecting the available cross-scale annotation coverage in the MH-SoyaHealthVision dataset. Future dataset collection efforts should prioritize UAV annotation across a broader range of disease categories, including Septoria Brown Spot and Frogeye Leaf Spot which were excluded from cross-domain experiments in this study due to the absence of corresponding UAV annotations, to enable more comprehensive multi-scale disease monitoring across the full spectrum of soybean health conditions. Finally, future work with access to higher-memory GPU hardware should investigate whether larger input resolutions beyond 224*×*224 recover additional diagnostic spatial detail for UAV-scale disease classification, as hardware constraints prevented evaluation of higher resolutions in the current study despite empirical attempts.

Overall, this multi-scale supervised contrastive framework provides a practical foundation for cross-domain soybean disease monitoring in precision agriculture settings.

## Data Availability

All data generated or analysed during this study are included in this published article.
